# Non-invasive and minimally invasive glucose monitoring devices: a systematic review and meta-analysis on diagnostic accuracy of hypoglycaemia detection

**DOI:** 10.1186/s13643-021-01644-2

**Published:** 2021-05-10

**Authors:** Nicole Lindner, Aya Kuwabara, Tim Holt

**Affiliations:** 1 Nuffield Department of Primary Care Health Sciences, University of Oxford, Oxford, OX2 6GG UK; 2 Department of Family Medicine, University of Marburg, Karl-von Frisch-Straße 4, 35043 Marburg, Germany

**Keywords:** Hypoglycemia, Diabetes mellitus, Blood glucose self-monitoring, Meta-analysis, Diagnostic accuracy

## Abstract

**Background:**

The use of minimally and non-invasive monitoring systems (including continuous glucose monitoring) has increased rapidly over recent years. Up to now, it remains unclear how accurate devices can detect hypoglycaemic episodes. In this systematic review and meta-analysis, we assessed the diagnostic accuracy of minimally and non-invasive hypoglycaemia detection in comparison to capillary or venous blood glucose in patients with type 1 or type 2 diabetes.

**Methods:**

Clinical Trials.gov, Cochrane Library, Embase, PubMed, ProQuest, Scopus and Web of Science were systematically searched. Two authors independently screened the articles, extracted data using a standardised extraction form and assessed methodological quality using a review-tailored quality assessment tool for diagnostic accuracy studies (QUADAS-2). The diagnostic accuracy of hypoglycaemia detection was analysed via meta-analysis using a bivariate random effects model and meta-regression with regard to pre-specified covariates.

**Results:**

We identified 3416 nonduplicate articles. Finally, 15 studies with a total of 733 patients were included. Different thresholds for hypoglycaemia detection ranging from 40 to 100 mg/dl were used. Pooled analysis revealed a mean sensitivity of 69.3% [95% CI: 56.8 to 79.4] and a mean specificity of 93.3% [95% CI: 88.2 to 96.3]. Meta-regression analyses showed a better hypoglycaemia detection in studies indicating a higher overall accuracy, whereas year of publication did not significantly influence diagnostic accuracy. An additional analysis shows the absence of evidence for a better performance of the most recent generation of devices.

**Conclusion:**

Overall, the present data suggest that minimally and non-invasive monitoring systems are not sufficiently accurate for detecting hypoglycaemia in routine use.

**Systematic review registration:**

PROSPERO 2018 CRD42018104812

**Supplementary Information:**

The online version contains supplementary material available at 10.1186/s13643-021-01644-2.

## Background

Hypoglycaemia is a common side effect of diabetes treatment. On average, a patient with type 1 diabetes has two episodes of symptomatic hypoglycaemia per week and experiences 1.0 to 1.7 episodes of severe hypoglycaemia per year [1, 2]. The consequences of hypoglycaemia do not just include the immediate symptoms and mortality [3], hypoglycaemic events also have an enormous impact on the long-term outcome (increased cardiovascular risk, impaired cognitive function) [4, 5]. Therefore, current guidelines recommend that patients with type 1 diabetes self-monitor their blood glucose (SMBG) 4–10 times a day [6]. However, the adherence to SMBG via glucometer was reported to be as low as 44% for adults with type 1 diabetes and 24% for adults with type 2 diabetes [7, 8]. Minimally (MID) and non-invasive devices (NID) aim to facilitate diabetes control and improve patients’ adherence. With hypoglycaemia being one of the most threatening complications of diabetes mellitus, it is critical that these devices are capable of accurately detecting hypoglycaemic episodes, especially in those patients who are unaware of their hypoglycaemic episodes. Comparison of different devices and between different studies is challenging as there is no consensus on how to optimally assess the general accuracy over the whole glycaemic range and the binary accuracy of hypoglycaemia detection of MID and NID [9]. Consequently, studies report diagnostic accuracy in many different ways (e.g. sensitivity/specificity, MARD (mean absolute relative difference)), which are often not directly comparable to each other and/or of uncertain clinical relevance.

While many manufacturers of MID and NID advertise the safety and convenience with which those devices warn of hypoglycaemic episodes, there is no clear evidence how accurately they can actually detect hypoglycaemia. Therefore, in this systematic review, we aim to assess the diagnostic accuracy of hypoglycaemia detection of MID and NID.

## Methods

The study protocol for this review was registered on PROSPERO on 27/07/2018 (CRD42018104812).

### Data sources and searches

A literature search was conducted in June 2018 using the following databases: Clinical Trials.gov, Cochrane Library, Embase, PubMed, ProQuest, Scopus and Web of Science. Search phrases used for the search are given in supplement 1. These were reviewed with a healthcare librarian (NR) specialised in planning systematic reviews. We did not apply any language restriction. The references of included articles were scanned and the “related articles” feature in PubMed was used. We contacted manufacturers of MID and NID to seek unpublished data. To screen for newly published articles, we performed two updated searches (29th of March 2019 and 19th of December 2019). To search for articles investigating diagnostic accuracy of recently released devices, we additionally performed a pragmatic search on 26th of October 2020 in PubMed.

### Study selection

We included any prospective, clinical diagnostic test accuracy study including children or adults with type 1 diabetes or type 2 diabetes, where MID or NID was compared to venous, capillary or arterial blood as a reference standard. Studies with only a sub-group eligible for inclusion were also included. The target condition was hypoglycaemia, determined by biochemical criteria with a glucose concentration of at least ≤100 mg/dl. Studies investigating different thresholds at the same time were also included. Studies eligible for inclusion should provide sufficient information on sensitivity and specificity of hypoglycaemia detection. Excluded were retrospective simulated data analyses of pre-existing data sets, in vitro studies, in vivo studies in species other than human and studies in participants with other types of diabetes (e.g. gestational diabetes or cystic fibrosis-related diabetes).

### Data extraction

Two reviewers (NL and AK) independently assessed the eligibility of identified articles in a two-step approach ((1) abstract and title screening, (2) full-text screening). Endnote X5 and X8 (Clarivate Analytics, PA, USA) and Excel 2016 (Microsoft, Redmond, WA, USA) were used to catalogue the results. Disagreements among reviewers were resolved through consensus. The study selection process was reported in a Preferred Reporting Items for Systematic Reviews and Meta-Analyses (PRISMA) flow diagram. Two reviewers (NL and AK) independently extracted data using a standardised data extraction form (supplement 3). Outstanding data were sought by a pre-specified procedure (two e-mails separated by a time interval of 2 weeks to the corresponding author).

### Quality assessment

Two reviewers (NL and AK) independently assessed the quality of included studies using a review-tailored Quality Assessment of Diagnostic Accuracy Studies 2 (QUADAS-2) tool [10]. Disagreements among reviewers were resolved through consensus. The outcome of the methodological quality assessment was presented in two tables, showing the individual study with their risk of bias in each of the four domains and a summary graph of all of studies. The tables were created using the Review Manager 5 Software [11]. The risk of bias was explored in sensitivity analyses by excluding studies with overall high risk of bias. The overall risk of bias was rated as high when two domains of the QUADAS-2 tool were at high risk of bias.

### Data synthesis and analysis

For each study, contingency tables of hypoglycaemia detection comparing index test to reference standard were constructed and sensitivity and specificity for each study were calculated. If authors had performed diagnostic accuracy analysis for multiple thresholds, the main analysis was performed using the threshold value most commonly employed among the included studies. As data for multiple thresholds were available, we additionally analysed diagnostic accuracy with regard to the level of glucose (basis of this analysis was the hypoglycaemia definition of the American Diabetes Association, which defines a blood glucose value equal to or below 70 mg/dl (3.9 mmol/l) as hypoglycaemic) [4]. If data for more than one reference standard were available, the superior reference standard (venous blood and not capillary blood) was used for the main analysis. If data for more than one insertion site was given, the data on the officially approved insertion site was used for the main analysis.

To calculate pooled estimates for meta-analysis, the bivariate random effects model of Reitsma et al. [12] implemented in the madad package [13] for R software for statistical computing [14] was used. Paired forest plots and hierarchical summary receiver operating characteristic (SROC) curves were drawn using metaplot version 0.4 [15]. The magnitude of heterogeneity was visually examined in SROC curves and forest plots as recommended by the Cochrane Collaboration [16]. In addition, the effects of pre-specified covariates were explored via meta-regression and sub-group analyses. Sensitivity and specificity were calculated individually for pre-specified sub-groups and a likelihood-ratio test was used to assess the difference of sub-groups. The primary analysis included all eligible studies. To prove the robustness of findings, we excluded studies with high overall risk of bias according to the QUADAS-2 tool in the sensitivity analysis. Tests for funnel plot asymmetry have low power to detect publication bias in diagnostic accuracy studies when there is considerable heterogeneity and were therefore not performed [16–18].

## Results

### Literature search led to inclusion of 14 studies

**Fig. 1:**
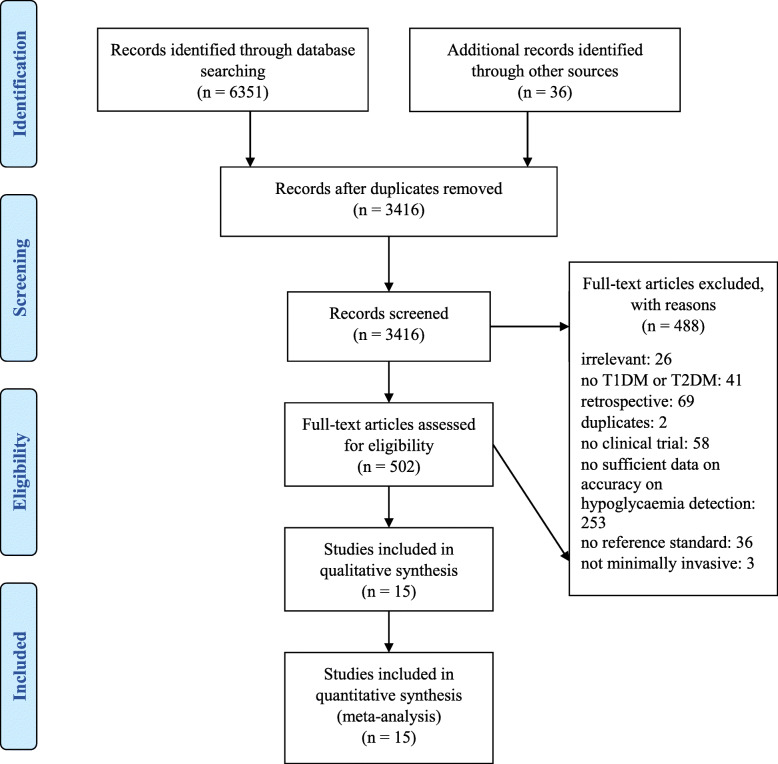
PRISMA flow diagram showing results of the screening. A total of 3416 nonduplicate results were identified and the full text of 502 was assessed. This led to an inclusion of 14 articles containing 15 studies

The search was performed in December 2019 and identified 3416 nonduplicate results. Of those, 502 articles were identified as eligible by abstract and title screening and 14 articles containing 15 studies were included after the full-text screening. Figure 1 shows the PRISMA flow diagram. Supplement 2 gives an overview of reasons of the exclusion of studies which partly fulfilled inclusion criteria.

### Details of included studies

Fourteen articles including 15 studies with a total of 733 patients were included into the final analysis. Characteristics of those studies are shown in Table 1. Eight studies were performed in North America, six in Europe and one in Asia. Most of the trials investigated the diagnostic accuracy of MID (MID: 13 studies, NID: 2 studies). Seven studies used capillary blood as the only reference standard test, six compared MID or NID to capillary and venous blood and two studies had venous blood as the only reference standard test. Eight studies addressed diagnostic accuracy in individuals with type 1 diabetes only, and in two of the included studies, just a sub-group of participants had diabetes. Different thresholds ranging from 40 to 100 mg/dl for hypoglycaemia detection were used. The most common threshold was 70 mg/dl (10 studies), which corresponds to the hypoglycaemia definition of the American Diabetes Association [4]. Three studies investigated diagnostic accuracy at different thresholds simultaneously. The mean age of participants ranged from 9.6 to 61.6 years and three studies included children. Laffel et al. encompass two independent trials. Therefore, the two trials were included separately: Laffel 2016, study 1, corresponds to the trial investigating diagnostic accuracy of the CGM (continuous glucose monitoring) G4 Platinum with its regular algorithm, whereas Laffel 2016, study 2, corresponds to the trial investigating diagnostic accuracy of G4 Platinum with a modified (Software 505) algorithm [21].

**Table 1:** Summary of the included studies investigating the accuracy of hypoglycaemia detection of MID and NID

Device	Author, year	Country	Technique	Number of participants/paired measurements	Reference test	Type of diabetes	Setting	Threshold (mg/dl)	Mean age (years)/inclusion of children
Eversense (Senseonics)	Christiansen, 2018 [19]	USA	MID	74/16653	Venous blood	T1DM: 67.8% T2DM: 29%	Hospital	70	45.1/no
G4 Platinum (Dexcom)	Steineck, 2019 [20]	Denmark	MID	14/681 (venous)	Capillary and venous blood	T1DM: 100%	Hospital	70	48/no
G4 Platinum, regular algorithm (Dexcom)	Laffel, 2016 Study 1 [21]	USA	MID	176/2922	Venous and capillary blood	T1DM: 99% T2DM: 1%	Hospital	80	11.4/yes
G4 Platinum, Software 505 algorithm (Dexcom)	Laffel, 2016 Study 2 [21]	USA	MID	79/2262	Venous and capillary blood	T1DM: 100%	Home	80	12.2/yes
G4 Platinum (Dexcom)	Bailey, 2015 [22]	USA	MID	51/2236	Venous and capillary blood	T1DM: 86% T2DM: 14%	Hospital and home	70	46.7/no
G4 Platinum (Dexcom)	Nakamura, 2015 [23]	USA	MID	68/9152 (venous)	Venous and capillary blood	T1DM: 83% T2DM: 17%	Home and hospital	100, 90, 80, 70	42.2/no
Guardian (Medtronic)	Bay, 2013 [24]	Denmark	MID	72/1786	Venous blood	T1DM: 100%	Hospital	72, 54, 40	no/55
Guardian (Medtronic)	Zijlstra, 2013 [25]	Germany	MID	18/2317	Capillary blood	T1DM: 100%	Hospital	70	43/no
Guardian (Medtronic)	Bode, 2004 [26]	USA	MID	68/4435	Capillary blood	T1DM: 100%	Home	70	44/no
CGMS Gold (Medtronic)	Lee, 2012 [27]	South Korea	MID	12/122	Capillary blood	DM: 41.67% Percentage of T1DM and T2DM not given	Hospital	70	50.6/no
CGMS Gold (Medtronic)	Adolfsson, 2009 [28]	Sweden	MID	12/182	Capillary blood	T1DM: 100%	During dive	70	31/no
CGMS (Medtronic)	Guerci, 2003 [29]	France	MID	18/ng	Capillary blood	T1DM: 100%	Hospital	55	40.4/no
STS (Dexcom)	Rabiee, 2009 [30]	USA	MID	19/1065 (capillary)	Capillary and venous blood	DM: 64.21% hereof 100% T2DM	Hospital	55	61.6/no
GlucoWatch (Cygnus)	Hathout, 2005 [31]	USA	NID	30/327	Capillary blood	T1DM: 100%	Home	100, 90, 80, 70	9.96/yes
Teledyne Sleep Sentry (Teledyne Avionics)	Johansen, 1986 [32]	Denmark	NID	22/99	Capillary blood	DM: 100% Percentage of T1DM and T2DM not given	Hospital	54	37/no

Laffel 2016, study 1, corresponds to the trial investigating the accuracy of the CGM G4 Platinum with its regular algorithm, whereas Laffel 2016, study 2, corresponds to the trial investigating the accuracy of G4 Platinum with a modified (Software 505) algorithm. *ng* not given

### Methodological quality of included studies was often insufficient

**Fig. 2:**
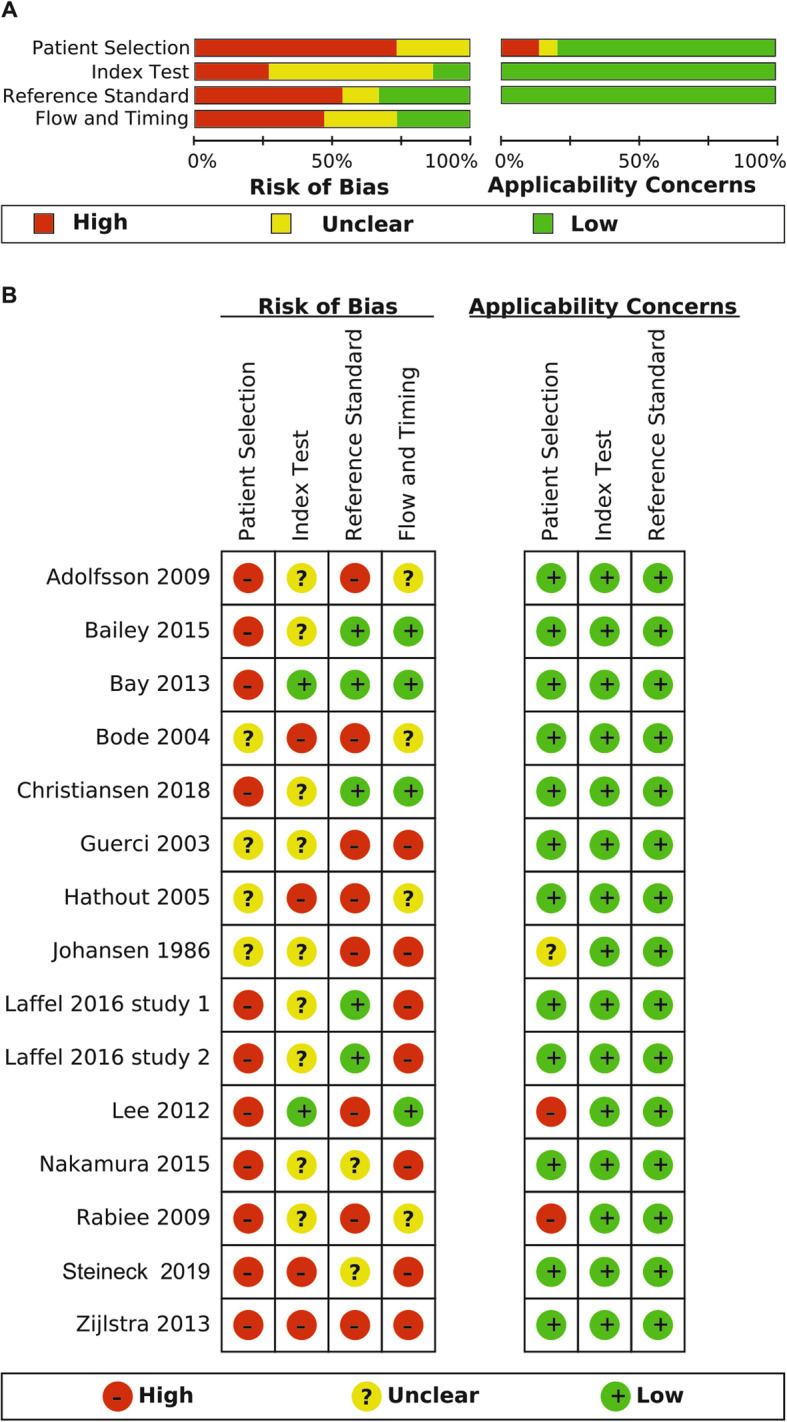
**a** Risk of bias graph. **b** Risk of bias summary. Methodological quality was assessed on four key domains (1. patient selection, 2. index test, 3. reference standard and 4. flow and timing). Therein none of the studies was assessed as low risk of bias in all of the four key domains. Applicability concerns were assessed in three key domains (1. patient selection, 2. index test, 3. reference standard) with the QUADAS-2 tool. Applicability concerns were generally lower

The methodological quality of the included studies was assessed in four key domains ((1) patient selection, (2) index test, (3) reference standard and (4) flow and timing) using the established Quality Assessment of Diagnostic Accuracy Studies (QUADAS) 2 tool [10]. Figure 2 summarises the overall risk of bias and applicability concerns. In general, across all of the studies, the methodological quality was often classified as either insufficient or unclear. (1) With regard to patient selection, the risk of bias was generally *unclear* or *high* as most of the studies included non-random series of participants or excluded participants inappropriately. (2) Regarding the risk of bias for the index test, only two studies were rated as *low risk* of bias. Looking at the other studies, insufficient information or biased interpretation of the reference standard led to classification as *unclear* or *high risk* of bias. (3) The risk of bias for the reference standard test was rated as *high* in eight studies because of the use of an inferior reference standard (capillary instead of venous blood) or interpretation of the reference standard with knowledge on the index test result. (4) In general, the risk of bias with regard to the flow of timing was high because of an inappropriate interval between the index test and reference standard.

However, applicability concerns were generally lower as all of the studies included patients with type 1 or type 2 diabetes, and all of the studies investigated the detection of hypoglycaemia in MID or NID defined by an acknowledged reference standard. With regard to the patient selection, applicability concerns were *high* in two of the studies as only a sub-group of participants had diabetes and *unclear* in one study as there was not enough information provided on the participants.

### MID and NID had a pooled mean sensitivity of 69.3% and a mean specificity of 93.3%

**Fig. 3:**
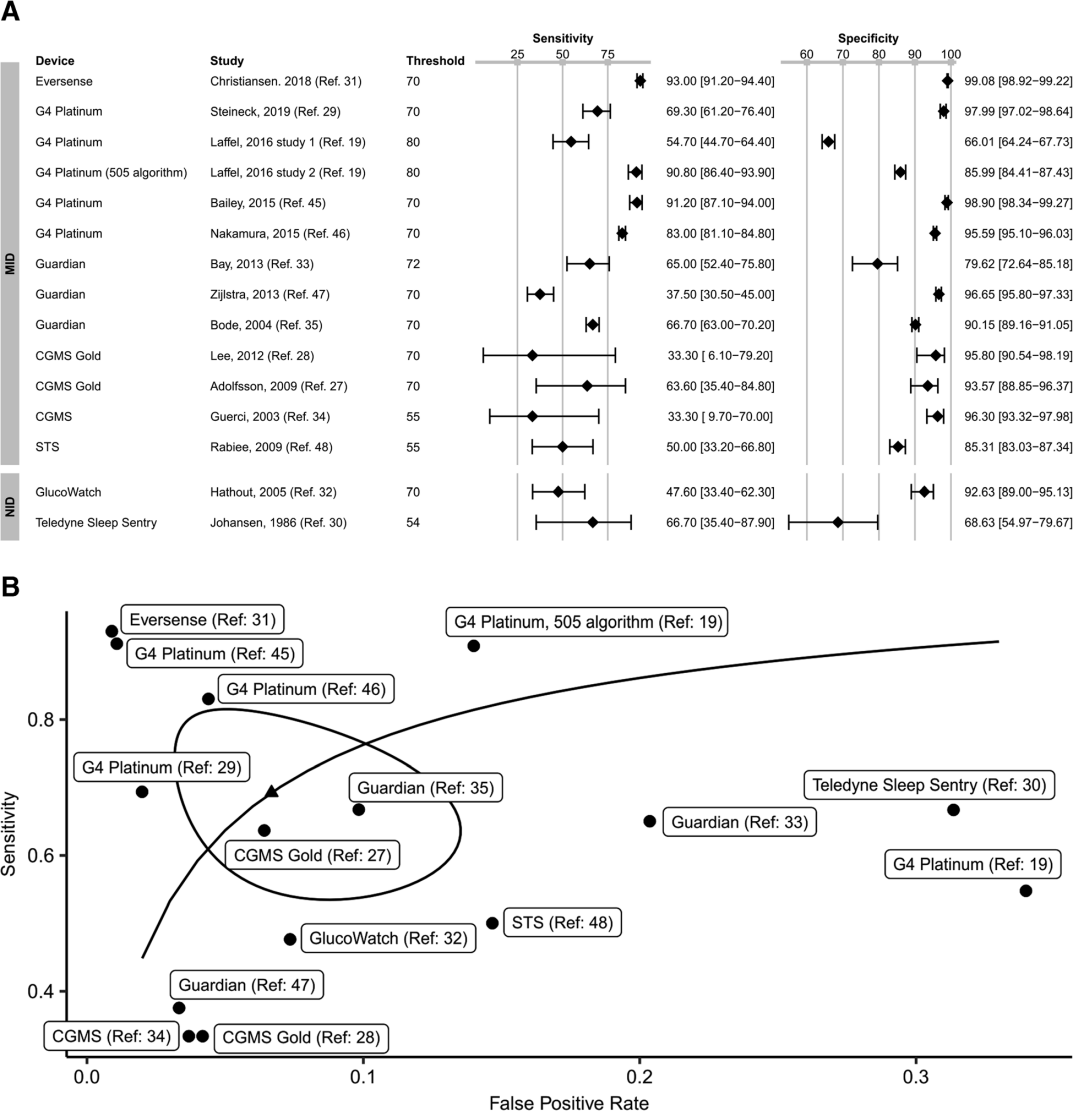
**a** Forest plot of sensitivity and specificity with 95% confidence interval in brackets of minimally invasive devices (MID) and non-invasive devices (NID) for detection of hypoglycaemia in each study. Pooling all of the studies resulted in a mean sensitivity of 69.3% [95% CI: 56.8 to 79.4] and a mean specificity of 93.3% [95% CI: 88.2 to 96.3], *n* number of participants, threshold in mg/dl. **b** Summary receiver operating characteristic (SROC) curve for overall diagnostic accuracy to detect hypoglycaemia of MID and NID. SROC, solid curve; individual studies, circles; summary estimate, triangle; 95% confidence region, contour ellipsoid

Pooling the data resulted in a relatively low mean sensitivity of 69.3% [95% CI: 56.8 to 79.4] and a mean specificity of 93.3% [95% CI: 88.2 to 96.3]. Diagnostic accuracy showed a great variation reflecting that in individual studies, sensitivity varied between 33.3 and 91% and specificity between 66 and 98.9%. Figure 3 displays the paired forest plot of sensitivity and specificity and the resulting summary receiver operating characteristic (SROC) curve.

### Impaired diagnostic accuracy in the hypoglycaemic range of latest generation devices

The field of continuous glucose monitoring (CGM) is rapidly evolving, and devices with advanced techniques and algorithms are introduced regularly. Several studies investigating the latest generation devices were found in our systematic literature screening. However, none of those provided the data necessary to determine diagnostic accuracy in the hypoglycaemic range in terms of sensitivity and specificity [33–36]. This impeded formal inclusion of those studies into the meta-analysis. To avoid losing the valuable information contained in these studies, we moved on to perform an explorative sub-analysis on any data on diagnostic accuracy in the hypoglycaemic range provided in those studies: Wadwa et al. in their study report a missed detection rate of 26% and a false alert rate of 30% for hypoglycaemia < 60 mg/dl for the Dexcom G6 [37]. In the adult study population of Alva et al., FreeStyle Libre 2 missed 24% of the hypoglycaemic events (< 60 mg/dl) and 28% of alarms were false [38]. Szadkowska et al. investigated the diagnostic accuracy of Free Style Libre with new glucose algorithm measurement in children. They state that accuracy was best in stable glycaemic conditions and deteriorated significantly when glucose was falling abruptly. Furthermore, they report a significant tendency of Free Style Libre 2 FSL to overestimate blood glucose. Therefore, they recommend to double-check CGM values with SMBG measurement in hypoglycaemia [39]. Table 2 provides an overview on data on diagnostic accuracy in the whole glycaemic range and in hypoglycaemia of more recent devices.

**Table 2:** Summary of studies investigating the accuracy of more recent MIDs in the hypoglycaemic range

Device (study)	Population	General accuracy (*n*)	Accuracy in hypoglycaemia (*n*)
Dexcom G6 (Castorino, 2020) [40]	32 pregnant participants with T1DM, T2M, GDM	Overall MARD 10.3% (734)	54–69 mg/dl MAD 6.9 mg/dl 79.2% in %15/15 (24) 40–53 mg/dl MAD 7.9 mg/dl 100% in %15/15 (8)
Dexcom G6 (Welsh, 2019) [41]	49 participants with T1DM	Overall MARD 7.7% (1387)	< 70 mg/dl MARD 13.3% MAD 9.1 mg/dl 81.5% in %15/15 (81)
Dexcom G6 (Shah, 2018) [42]	Reprocessed data of 76 participants with T1DM and T2DM	Overall MARD 9.0% (3532)	< 70 mg/dl MARD 9.5% 80% in %15/15 (185)
Free Style Libre 2 (Alva, 2020) [38]	273 participants with T1DM and T2DM	Overall MARD 9.2% (adults), 9.7% (children) (25510)	< 70 mg/dl Adults: 94.3% in %15/15 (3473) 89.3% true detection rate, 86% true alarm rate Children: 96.1% in %15/15 (882)
Eversense and Freestyle Libre (Fokkert, 2020) [43]	23 participants with T1DM	Eversense: MARD 17% (exercise), 13% (normal daily activity) Freestyle Libre: MARD 20% (exercise), 12% (normal daily activity) (1722)	< 70 mg/dl Eversense: > 85% within ±15 mg/dl 72% (exercise) 76% (normal daily activity) Freestyle Libre: > 85% within ±15 mg/dl 61% (exercise) 78% (normal daily activity)

*T1DM* diabetes type 1, *T2DM* diabetes type 2, *GDM* gestational diabetes mellitus, *n* number of matched pairs, *MARD* mean absolute relative difference, *MAD* mean absolute difference in mg/dl

### Meta-regression analysis shows that heterogeneity is explained by 4 covariates

Next, we investigated if the studied index test technique (MID vs. NID) influenced sensitivity and specificity. MID were more often studied than NID: 13 studies investigated the diagnostic accuracy of MID while NID were only assessed in two of the included studies. For MID, sensitivity and specificity varied greatly and pooling the studies resulted in a mean sensitivity of 71.1% [95% CI: 57.6 to 81.7] (range 33.3 to 93) and a mean specificity of 94.2% [95% CI: 89.3 to 96.9] (range 66 to 99.1). Two studies assessed the performance of NID (sensitivity: Hathout et al. [31], 48% [95% CI: 33 to 62] and Johansen et al. [32], 67% [95% CI: 35 to 88]; specificity: Hathout et al. [31], 93% [95% CI: 89 to 95] and Johansen et al. [32], 69% [95% CI: 55 to 80]). Those are the only two studies included investigating the diagnostic accuracy of NID. Moreover, these NIDs are not commercially available anymore. Thus, the validity of a comparison of MID vs. NID is limited. Figure 3b summarises the study results colour coded by technique.

**Fig. 4:**
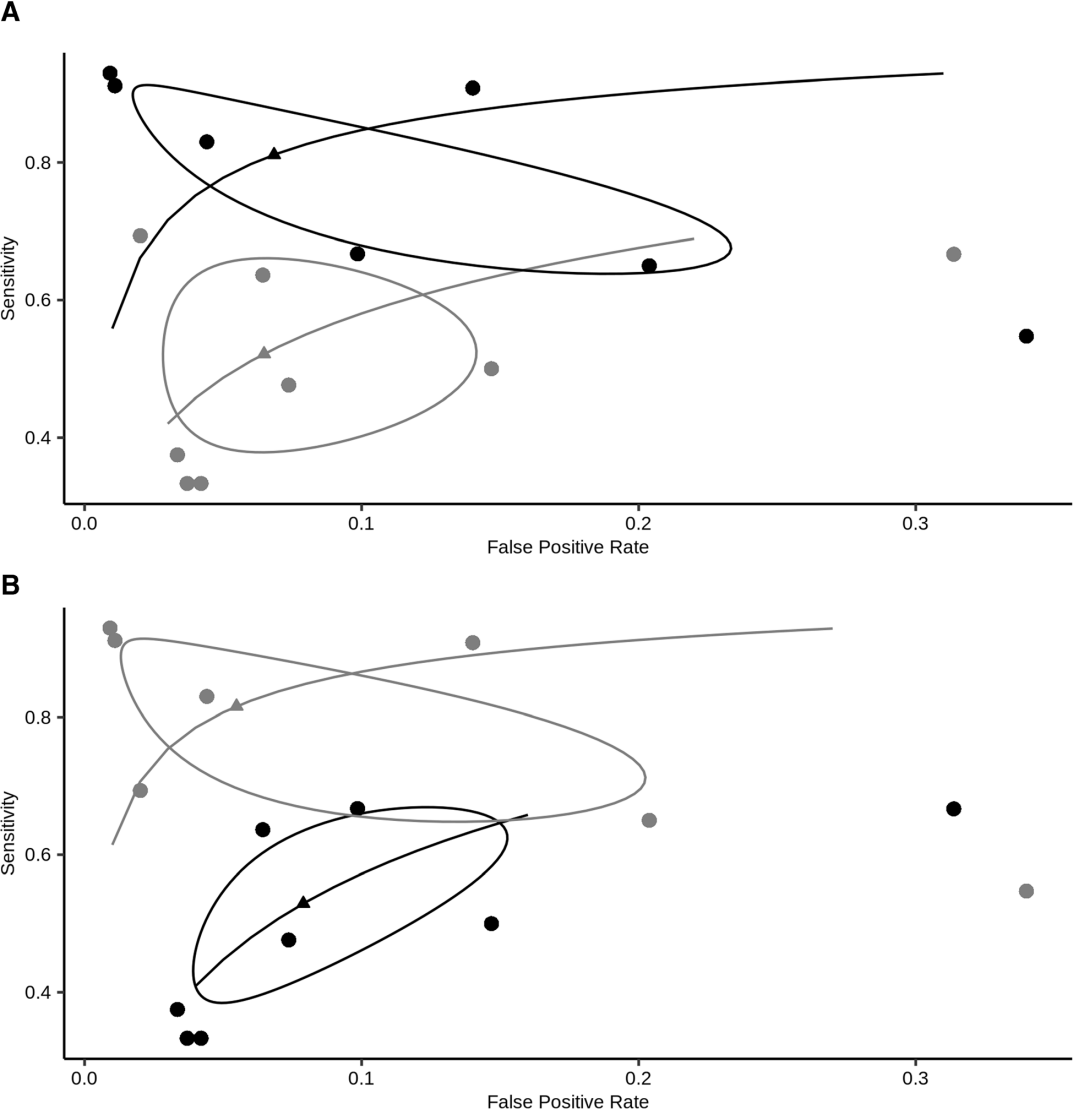
**a** Summary ROC for MID and NID to detect hypoglycaemia (SROC curve, solid curve; study data, circles; summary estimate, triangle; 95% confidence region, contour ellipsoid; venous blood as the reference standard, grey; capillary blood as the reference standard, black). Pooled sensitivity was significantly higher in trials using venous blood as the reference standard, whereas the influence on pooled specificity was not significant. **b** Summary receiver operating characteristic (SROC) curve for overall diagnostic accuracy to detect hypoglycaemia of MID and NID. SROC, solid curve; study data, circles; summary estimate, triangle; 95% confidence region, contour ellipsoid; high number of participants (> 50), black; low number of participants (≤50), grey. The pooled sensitivity was significantly higher in studies investigating a larger study cohort

The included studies compared NID or MID to different reference standards. Seven studies used capillary blood as the only reference standard, six compared MID or NID to capillary and venous blood and two studies had venous blood as the only reference standard. Studies using venous blood as the reference standard indicated a higher sensitivity than studies using capillary blood as the reference standard (venous 81.6% [95% CI: 68.7 to 89.9] vs. capillary 52.9% [95% CI: 41.3 to 64.3], *p*-value: < 0.001***). Yet, a significant difference in specificity could not be observed (venous 94.5% [95% CI: 84.1 to 98.3] vs. capillary 92.1% [95% CI: 86.6 to 95.5], *p*-value: 0.55). The likelihood-ratio test confirmed the result (*χ*^2^: 9.81, *p*-value: 0.007**). The corresponding SROC is displayed in Fig. 4a. As venous reference standard test, YSI (Yellow springs instrument, YSI Inc, OH, USA) was used in all studies except one (Bay et al. [24] used Hitachi, Roche, Basel, Switzerland), whereas a number of different devices were used as capillary reference standard (e.g. Accu-Chek (Roche), StatStrip Xpress (Nova Biomedical), OneTouch Ultra 2 meter (Onetouch)).

Most studies included a limited number of participants (2 studies investigated only 12 participants [27, 28] and one study only 14 participants [20]). Yet, the cohort of the largest study included 176 participants [21]. We investigated whether there is an association of study size with observed diagnostic accuracy. Indeed, the pooled sensitivity was higher in trials with a larger study cohort or multi-centre trials (larger study cohort (> 50 participants) or multi-centre: 81.1% [95% CI: 67.8 to 89.8] vs. low number of participants or single-centre: 52.2% [95% CI: 40.6 to 63.5], *p*-value: < 0.001***, *χ*^2^: 12.79, *p*-value: 0.00167**). There was no significant effect on pooled specificity. Corresponding SROC is given in Fig. 4b. There was also a high variability in the number of paired measurements. One study relied on only 99 paired measurements [32], while the highest number of paired measurements was 16,653 [19]. Analogous to the association of study size and sensitivity, the pooled sensitivity was higher in trials with a larger number of measurements (large number of measurements (≥1000): 74.8% [95% CI: 58.5 to 86.2] vs. small number of measurements: 58.8% [95% CI: 47.2 to 69.5], *p*-value: 0.207).

In many studies, in addition to sensitivity and specificity of hypoglycaemia detection, further parameters of accuracy were reported. Ten studies described accuracy in terms of mean absolute relative difference (MARD), which is a parameter that shows overall device accuracy over the whole glycaemic range. In trials showing a better overall accuracy expressed in a lower MARD, the mean sensitivity was higher (low MARD (≤ 10%): 92% [95% CI: 89.9 to 93.6] vs. high MARD: 61.7% [95% CI: 48.2 to 73.6], *p*-value: < 0.001***, *χ*^2^: 12.38, *p*-value: 0.00205**). An insignificant difference of mean specificity was also observed (low MARD: 97.5% [95% CI: 86.3 to 99.6] vs. high MARD: 93.2% [95% CI: 86.4 to 96.8], *p*-value: 0.216). Corresponding SROC is given in supplement 4. In this context, also other parameters of accuracy, like the correlation coefficient and percentage of measurements in zones A and B of the Clarke Error Grid Analysis, showed a similar relationship with pooled sensitivity and specificity.

Covariates relating to the study setting were analysed. Here (1) artificial adjustment of blood glucose, (2) funding by manufacturers and (3) age of the study showed an influence on diagnostic accuracy. (1) Artificial adjustment of blood glucose via insulin administration (“insulin challenge”) was associated with a highly significant increase of pooled sensitivity (insulin administration: 85.6% [95% CI: 72 to 93.2] vs. no insulin administration: 55.6% [95% CI: 45.5 to 65.2], *p*-value: < 0.001***). An association with specificity was not found (insulin administration: 95% [95% CI: 80.7 to 98.9] vs. no insulin administration: 93.5 % [95% CI: 89.3 to 96.1], *p*-value: 0.693). (2) Furthermore, in studies funded by manufacturers, there was a significant difference of pooled sensitivity (manufacturer-funding: 82% [95% CI: 65.9 to 91.5] vs. no-manufacturer-funding: 59.2% [95% CI: 44 to 72.9], *p*-value: 0.031*, *χ*^2^: 6.717, *p*-value: 0.0348*), while no influence on specificity was seen (manufacturer-funding: 92.5 [95% CI: 75 to 98.1] vs. no-manufacturer-funding: 93.8% [95% CI 88.5 to 96.8], *p*-value 0.793). (3) The age of the study showed a relationship with measured diagnostic accuracy. Newer studies revealed a non-significantly higher sensitivity (new studies: 75.8% [95% CI: 59.4 to 87] vs. old studies: 57.5% [95% CI: 45.9 to 68.3], *p*-value: 0.086) and a slightly higher specificity (new studies: 94.9% [95% CI: 88.2 to 97.9] vs. old studies: 90% [95% CI: 82.4 to 94.6], *p*-value: 0.258). The location of the study (hospital vs. other (home/outdoor)) did not have a significant influence on pooled sensitivity or specificity.

Interestingly, no association of participant characteristics (including mean age, gender, proportion of participants with type 1 diabetes and BMI) with pooled sensitivity and specificity was observed. Two studies also included participants that did not have diabetes (Lee et al. [27], 42% of participants had diabetes; Rabiee et al. [30], 64% of participants had diabetes). The sensitivity was notably lower in studies including patients without diabetes (pooled sensitivity: 42.2% [95% CI: 21.9 to 65.59] vs. 71.4% [95% CI: 58.3 to 81.6]), whereas there was no difference in specificity (pooled specificity: 91.5% [95% CI: 73.9 to 97.6] vs. 93.6% [95% CI: 87.8 to 96.7]).

The included studies employed different thresholds ranging from 40 to 100 mg/dl for hypoglycaemia detection. The most common threshold was 70 mg/dl (10 studies), which corresponds to the hypoglycaemia definition of the American Diabetes Association [4]. Only pooling data of studies applying the threshold recommended by the American Diabetes Association resulted in a slightly higher pooled sensitivity (71.1% [95% CI: 55.9 to 82.7]) and a slightly higher pooled specificity (95.8% [95% CI: 92.4 to 97.8]). Three studies investigated diagnostic accuracy for different thresholds simultaneously. Inclusion of these data in additional meta-regression analyses showed that, as expected, higher cut-off values were associated with increased sensitivity and decreased specificity. A corresponding forest plot is given in supplement 5.

To investigate whether the findings of this systematic review are robust, sensitivity analyses were undertaken. As occasionally the quality of included studies was unsatisfactory, the influence of studies of poor quality on the results was analysed: Exclusion of studies with high risk of bias according to the QUADAS-2 tool did not have a notable influence on sensitivity.

### Rate of device failure is reported as high

Additionally, the performance of different devices was analysed. Ten out of the 15 studies reported on sensor stability. All in all, the device failure rate is reported as high throughout the studies. In the study of Adolfsson et al., 42% of the participants needed a device replacement during the trial of three days duration [28]. However, as this study investigated the diagnostic accuracy of CGMS Gold (Medtronic) in the context of diving, this may underestimate the actual stability in a normal setting. Yet also, Hathout et al. report that 33% of the HypoMon measurements were unusable [31]. Reasons for the high rate of device malfunction are not always discussed, but calibration and transmission failures are reported.

### Side effects and adverse events are common

Furthermore, side effects and adverse events of different devices were analysed. Six out of the 15 studies reported on side effects. Two studies reported the occurrence of no side effects or adverse events [24, 29], whereas the rate of reported side effects was high in the other studies. The highest number of side effects was seen by Hathout et. al., where 35% of the participants withdrew because of side effects [31]. The studies from Christiansen et al. and Bode et al. reported both a similar rate of side effects of approximately 10% [19, 26]. Most of the side effects were instances of mild irritation, bleeding or discomfort. However, two more notable side effects were reported by Christiansen et al.: First, two events were described where a small element presumably has been translocated into the participant’s body. Those two events are rated as mild in severity due to small size and biocompatibility. Second, a device could not be removed in local anaesthesia as planned but general anaesthesia was required. This event was adjudicated as serious [19].

### Strengths and weaknesses of individual devices

In this presented work, some devices seemed to be more accurate than others. However, in addition to pure accuracy, other factors relating to the use of MID or NID might be important from the patient’s perspective. In this meta-analysis, Eversense (Senseonics, Inc., USA) revealed the highest sensitivity and specificity in detection of hypoglycaemic events. In contrast to other devices, Eversense can be used for relatively long periods (up to 90 days) and the transmitter can be removed and replaced. Calibration is needed twice daily. On the other hand, the sensor cannot be placed by the patients themselves but by a healthcare professional. The placement is more invasive than the procedure for other MID, and the rate of side effects of Eversense was higher and more serious compared to other MID. The second highest accuracy in detection of hypoglycaemic events was seen in Dexcom G4 Platinum (DexCom Inc, USA). However, contemplating the results of this meta-analysis, diagnostic accuracy showed a great variation (sensitivity ranged from 54.7 to 91.2 %). The sensor of this particular device can be worn for up to 7 days, calibration is needed twice daily and the sensor can be placed by the patients themselves. The sensor stability seems to be satisfactory and the rate of side effects seems to be low.

## Discussion

In this work, we provide a comprehensive review and meta-analysis on the diagnostic accuracy of MID and NID for hypoglycaemia detection in patients with type 1 diabetes and type 2 diabetes. Fifteen studies with a total of 733 participants evaluating the diagnostic accuracy of hypoglycaemia detection of MID and NID were included. The mean sensitivity was 69.3% and the mean specificity was 93.3%. There was remarkable heterogeneity among the included studies. Meta-regression analyses revealed an association of type of reference standard test (venous vs. capillary blood), number of participants, reported overall performance, artificial manipulation of blood glucose and funding by manufacturers with device performance in hypoglycaemia detection. Pooled sensitivity was significantly higher in studies funded by device manufactures. Different reasons might contribute to this association. The study design might have been more rigorous in trials funded by manufacturers. This concept is supported by the fact that the sample size was generally higher and venous blood was used more often as the reference standard in those studies. On the other hand, in manufacturer-funded studies, trial protocols might have been chosen that tend to overestimate device performance. And indeed, induced hypoglycaemia by insulin administration was more commonly performed in these studies.

Additionally, we found that there is a notable rate of side effects and adverse events (in one case even a serious side effect). Furthermore, the sensor stability was reported as relatively poor throughout the studies.

While this work, to the best of our knowledge, for the first time reviews systematically the accuracy of MID and NID in detection of hypoglycaemia, a recent non-systematic review also sees limitations in the diagnostic accuracy of MID and NID and raises concerns regarding the frequency of false-positive alarms [44]. Interestingly, Howsmon et al. praise the high sensor accuracy and alarm sensitivity of CGM systems in their non-systematic review [45]. A reason for this discordant conclusion might be the fact that the authors make the assumption that an improved sensor accuracy in the hypoglycaemic range can be translated into providing more accurate hypoglycaemic alarms, which might not always follow. Notably, the authors of the UK recommendation on one particular, currently very popular device (FreeStyle Libre) are aware of these limitations as they recommend to validate hypoglycaemic values measured with FreeStyle Libre via finger-prick blood glucose testing [46].

Even though the present review reveals that an accurate detection of hypoglycaemic events can likely not be achieved with MID and NID, a recent meta-analysis has found that patients using MID spend less time in hypoglycaemia than patients using SMBG [47]. This finding could be due to reduced detection of hypoglycemic events; however, other reasons may lead to a reduction of time spent in hypoglycaemia, for example because users may be able to recognise a trend towards hypoglycaemia and take precautionary steps accordingly.

Interestingly, Koziel et al. found in their non-systematic review that this reduction of time in hypoglycaemia does not correlate with device accuracy in terms of MARD. However, in keeping with our findings, they reported a significant relationship between MARD and the detection of hypoglycaemic events [48].

### Implications for clinical practice

The aim of MID and NID is the accurate and user-friendly monitoring of glucose levels. The results of this review indicate that most devices are not yet able to detect hypoglycaemia with sufficient accuracy. In 1 year of using an average MID or NID, according to the results of this meta-analysis, a patient with type 1 diabetes is expected to experience about 17 false-positive alarms and about 32 false-negative measurements. Underlying this estimate is an incidence of two episodes of symptomatic hypoglycaemia per week per patient [1, 2]. The high number of false-positive alarms (especially during the night) may lead to user frustration, alarm fatigue and cessation of device use. Even worse, subsequent alarms may not be taken seriously and true hypoglycaemic events may be missed. The number of false-negative events is equally concerning, as a missed hypoglycaemic episode may be a life-threatening event. This is especially problematic when MID and NID do not confirm hypoglycaemia in the presence of related symptoms, especially during rapid changes in glucose levels [49]. This increases the risk of delayed hypoglycaemia detection. Therefore, based on the available data, MID and NID do not appear to be sufficiently accurate to replace SMBG for the detection of hypoglycaemic episodes on its own. Values measured via MID or NID in or near the hypoglycaemic range should be double-checked with another method (e.g. capillary blood).

### Implications for future research

As we also observed a lack of robust high-quality studies, larger and methodologically optimised works are needed to assess the accuracy of hypoglycaemia detection of MID and NID. The risk of bias was specifically high in terms of patient selection. Future studies should take care of including the relevant population (e.g. people unaware of hypoglycaemia should not be excluded). Investigating the comparative diagnostic accuracy among MID and NID is highly challenging [50]. Studies in which all patients are tested with different devices or are randomly assigned to receive one or another device (direct comparative studies/head-to-head) are needed [51]. This systematic review was not designed to provide a complete overview on adverse events and device failure. However, our data are indicative of a high number of adverse events and system failures, and this is likely to be an underestimate as harms may be underreported [52]. Therefore, further studies investigating the actual number and severity of side effects, and analysis of the sensor stability as well as reasons for system failure are mandatory.

### Strengths and limitations

This systematic review provides the first comprehensive review of the current evidence on the diagnostic accuracy of MID and NID for the detection of hypoglycaemia. However, some limitations need to be considered: It is generally challenging to investigate the diagnostic accuracy of MID or NID. Therefore, the quality of articles in this field of research often appears imperfect. Frequently, the incomplete reporting in the included studies impeded the assessment of their methodological quality. In particular, there was uncertainty with regard to the index test and the patient selection. This might lead to an overestimation of the accuracy of hypoglycaemia detection of NID and MID by the present systematic review. On the other hand, MID/NID technology is continuously being improved; therefore, our review may demonstrate an underestimation of diagnostic accuracy compared to the most recent devices. However, meta-regression analyses have only revealed an insignificant trend regarding an influence of the year of publication on diagnostic accuracy.

## Conclusions

The present data show that MID and NID are not sufficiently accurate for detecting hypoglycaemia in type 1 diabetes and type 2 diabetes in routine use. The indicated diagnostic accuracy was associated with a variety of factors including the type of reference standard test, study size, general device performance, artificial manipulation of blood glucose and study funding source. Additionally, we saw a notable rate of side effects and adverse events and a limited sensor stability.

## Supplementary Information

**Additional file 1 Supplement 1:** Search Strategies. **Supplement 2:** Reason for exclusion of articles, which partly met inclusion criteria. **Supplement 3:** Data extraction sheet. **Supplement 4:** Summary ROC for MID and NID to detect hypoglycaemia for studies indicating a lower MARD compared to studies indicating a higher MARD. **Supplement 5:** Forest plot of sensitivity and specificity with 95% confidence interval of MID and NID for detection of hypoglycaemia in studies applying different thresholds simultaneously. All of the supplements are provides as word file (.txt).

## Data Availability

The datasets during and/or analysed during the current study available from the corresponding author on reasonable request.

## Abbreviations

AUC: Area under the curve
CGM: Continuous glucose monitoring
ISO: International Organisation of Standardisation
MARD: Mean absolute relative deviation
MID: Minimally invasive monitoring device
NID: Non-invasive monitoring device
NICE: National Institute for Health and Care Excellence
NPV: Negative predictive value
PPV: Positive predictive value
PRISMA: Preferred Reporting Items for Systematic Reviews and Meta-Analyses
PROSPERO: International Prospective Register of Systematic Reviews
QUADAS: Quality Assessment of Diagnostic Accuracy Studies
SMBG: Self-monitoring of blood glucose
SROC: Summary receiver operating characteristic
YSI: Yellow springs instrument
95% CI: 95% confidence interval
