# Supplementary material for: Non-invasive and minimally invasive glucose monitoring devices: a systematic review and meta-analysis on diagnostic accuracy of hypoglycaemia detection
Source: Syst Rev. 2021 May 10;10:145. doi: 10.1186/s13643-021-01644-2 (PMC8111899; doi:10.1186/s13643-021-01644-2)
Supplement: Supplementary file 1 — Additional file 1 Supplement 1: Search Strategies. Supplement 2: Reason for exclusion of articles, which partly met inclusion criteria. Supplement 3: Data extraction sheet. Supplement 4: Summary ROC for MID and NID to detect hypoglycaemia for studies indicating a lower MARD compared to studies indicating a higher MARD. Supplement 5: Forest plot of sensitivity and specificity with 95% confidence interval of MID and NID for detection of hypoglycaemia in studies applying different thresholds simultaneously. All of the supplements are provides as word file (.txt). [file 13643_2021_1644_MOESM1_ESM.docx]

**Supplements**

**Supplement 1**

**Search Strategies**

The initial search was conducted on 21/06/2018, the first update search was performed on the 29th of March 2019 and the second update search was performed on the 19^th^ of December 2019.

**1. Search Terms used of the search in the Cochrane Library**

| “continuous glucose monitor*“  CGM  “interstitial glucose monitor*”  “subcutaneous glucose monitor*”  “flash glucose monitor*”  “non-invasive glucose monitor*”  “non invasive glucose monitor*”  “non-invasive glucose monitor*”  “non-invasive Glucose Assessment”  “non-invasive glucose assessment”  “non invasive glucose assessment”  “Noninvasive glycaemia monitor*”  “non invasive glycaemia monitor*”  “non invasive glycaemia monitor*”  “minimally invasive glucose monitor*”  SugarBEAT  GlucoTrack  “Combo Glucometer”  “GlucoWise”  “Paradigm Veo”  “MiniMed“  “iPro Professional”  “Dexcom G4”  “Dexcom G5”  “FreeStyle Libre Flash”  “FreeStyle Navigator II”  “FreeStyle Libre Pro”  (combined with OR) | AND | Accuracy  Sensitivity  Specificity  “hypoglycemia detection”  “hypoglycaemia detection”  “hypoglycemic detection”  “hypoglycaemic detection”  “threshold alarm”  Hypo  “positive predictive value”  “negative predictive value”  “likelihood ratio”  “hypoglycaemia prediction”  “hypoglycaemia prediction  (combined with OR) |
| --- | --- | --- |

**Search Terms used for the search in CinicalTrials.gov**

| Condition: Hypoglycemia  Other Terms: Hypoglycaemia | Intervention:  “continuous glucose monitor*“  CGM  “interstitial glucose monitor*”  “subcutaneous glucose monitor*”  “flash glucose monitor*”  “non-invasive glucose monitor  “minimally invasive glucose monitor*”  (combined with OR) |
| --- | --- |

**Search Terms used for the search in Embase**

(no “related terms”)

| “continuous glucose monitor*“  CGM  “interstitial glucose monitor*”  “subcutaneous glucose monitor*”  “flash glucose monitor*”  “non-invasive glucose monitor*”  “non invasive glucose monitor*”  “non-invasive glucose monitor*”  “non-invasive Glucose Assessment”  “non-invasive glucose assessment”  “non invasive glucose assessment”  “Noninvasive glycaemia monitor*”  “non invasive glycaemia monitor*”  “non invasive glycaemia monitor*”  “minimally invasive glucose monitor*”  SugarBEAT  GlucoTrack  “Combo Glucometer”  “GlucoWise”  “Paradigm Veo”  “MiniMed“  “iPro Professional”  “Dexcom G4”  “Dexcom G5”  “FreeStyle Libre Flash”  “FreeStyle Navigator II”  “FreeStyle Libre Pro”  (combined with OR) | AND | Accuracy  Sensitivity  Specificity  “hypoglycemia detection”  “hypoglycaemia detection”  “hypoglycemic detection”  “hypoglycaemic detection”  “threshold alarm”  Hypo  “positive predictive value”  “negative predictive value”  “likelihood ratio”  “hypoglycaemia prediction”  “hypoglycaemia prediction  (combined with OR) |
| --- | --- | --- |

**Search Terms used for the search in PubMed**

| “continuous glucose monitor*“  CGM  “interstitial glucose monitor*”  “subcutaneous glucose monitor*”  “flash glucose monitor*”  “non-invasive glucose monitor*”  “non invasive glucose monitor*”  “non-invasive glucose monitor*”  “non-invasive Glucose Assessment”  “non-invasive glucose assessment”  “non invasive glucose assessment”  “Noninvasive glycaemia monitor*”  “non invasive glycaemia monitor*”  “non invasive glycaemia monitor*”  “minimally invasive glucose monitor*”  SugarBEAT  GlucoTrack  “Combo Glucometer”  “GlucoWise”  “Paradigm Veo”  “MiniMed“  “iPro Professional”  “Dexcom G4”  “Dexcom G5”  “FreeStyle Libre Flash”  “FreeStyle Navigator II”  “FreeStyle Libre Pro”  (combined with OR) | AND | Accuracy  Sensitivity  Specificity  “hypoglycemia detection”  “hypoglycaemia detection”  “hypoglycemic detection”  “hypoglycaemic detection”  “threshold alarm”  Hypo  “positive predictive value”  “negative predictive value”  “likelihood ratio”  “hypoglycaemia prediction”  “hypoglycaemia prediction  (combined with OR) |
| --- | --- | --- |

**Search Terms used for the Search in**

(limited to “Conference Papers & Proceedings”, “Dissertations and Theses”, search in abstract)

| “continuous glucose monitor*“  CGM  “interstitial glucose monitor*”  “subcutaneous glucose monitor*”  “flash glucose monitor*”  “non-invasive glucose monitor*”  “non invasive glucose monitor*”  “non-invasive glucose monitor*”  “non-invasive Glucose Assessment”  “non-invasive glucose assessment”  “non invasive glucose assessment”  “Noninvasive glycaemia monitor*”  “non invasive glycaemia monitor*”  “non invasive glycaemia monitor*”  “minimally invasive glucose monitor*”  SugarBEAT  GlucoTrack  “Combo Glucometer”  “GlucoWise”  “Paradigm Veo”  “MiniMed“  “iPro Professional”  “Dexcom G4”  “Dexcom G5”  “FreeStyle Libre Flash”  “FreeStyle Navigator II”  “FreeStyle Libre Pro”  (combined with OR) | AND | Accuracy  Sensitivity  Specificity  “hypoglycemia detection”  “hypoglycaemia detection”  “hypoglycemic detection”  “hypoglycaemic detection”  “threshold alarm”  Hypo  “positive predictive value”  “negative predictive value”  “likelihood ratio”  “hypoglycaemia prediction”  “hypoglycaemia prediction  (combined with OR) |
| --- | --- | --- |

**Search Terms used for the search in Scopus:**

| “continuous glucose monitor*“  CGM  “interstitial glucose monitor*”  “subcutaneous glucose monitor*”  “flash glucose monitor*”  “non-invasive glucose monitor*”  “non invasive glucose monitor*”  “non-invasive glucose monitor*”  “non-invasive Glucose Assessment”  “non-invasive glucose assessment”  “non invasive glucose assessment”  “Noninvasive glycaemia monitor*”  “non invasive glycaemia monitor*”  “non invasive glycaemia monitor*”  “minimally invasive glucose monitor*”  SugarBEAT  GlucoTrack  “Combo Glucometer”  “GlucoWise”  “Paradigm Veo”  “MiniMed“  “iPro Professional”  “Dexcom G4”  “Dexcom G5”  “FreeStyle Libre Flash”  “FreeStyle Navigator II”  “FreeStyle Libre Pro”  (combined with OR) | AND | Accuracy  Sensitivity  Specificity  “hypoglycemia detection”  “hypoglycaemia detection”  “hypoglycemic detection”  “hypoglycaemic detection”  “threshold alarm”  Hypo  “positive predictive value”  “negative predictive value”  “likelihood ratio”  “hypoglycaemia prediction”  “hypoglycaemia prediction  (combined with OR) |
| --- | --- | --- |

**Search Terms used for the search in Web of Science**

| “continuous glucose monitor*“  CGM  “interstitial glucose monitor*”  “subcutaneous glucose monitor*”  “flash glucose monitor*”  “non-invasive glucose monitor*”  “non invasive glucose monitor*”  “non-invasive glucose monitor*”  “non-invasive Glucose Assessment”  “non-invasive glucose assessment”  “non invasive glucose assessment”  “Noninvasive glycaemia monitor*”  “non invasive glycaemia monitor*”  “non invasive glycaemia monitor*”  “minimally invasive glucose monitor*”  SugarBEAT  GlucoTrack  “Combo Glucometer”  “GlucoWise”  “Paradigm Veo”  “MiniMed“  “iPro Professional”  “Dexcom G4”  “Dexcom G5”  “FreeStyle Libre Flash”  “FreeStyle Navigator II”  “FreeStyle Libre Pro”  (combined with OR) | AND | Accuracy  Sensitivity  Specificity  “hypoglycemia detection”  “hypoglycaemia detection”  “hypoglycemic detection”  “hypoglycaemic detection”  “threshold alarm”  Hypo  “positive predictive value”  “negative predictive value”  “likelihood ratio”  “hypoglycaemia prediction”  “hypoglycaemia prediction  (combined with OR) |
| --- | --- | --- |

**Supplement 2**

**Reason for exclusion of articles, which partly met inclusion criteria**

| **Article** | **Reason for exclusion** |
| --- | --- |
| (Adolfsson et al., 2011) | Aim of the study was to investigate whether minimally invasive glucose monitoring detects more hypoglycaemic episodes than frequent blood glucose measurements  authors provide information about the mean number of hypoglycaemic episodes detected be reference method and minimally invasive glucose monitoring but no direct comparison of the two methods with each other (e.g. 2*2 table) |
| (Alva, Bailey et al. 2020) | Data was not sufficient to calculate 2*2table (number of true positives differs whether calculated via detection rate or via true alarm rate, number in table 4 and 7 for measurements <70mg/dl does not fit) |
| (Bailey et al., 2011a) | Percentage of accurate and inaccurate readings in hypoglycaemic, euyglycaemic and hyperglycaemic range is reported, but no absolute numbers, therefore it is not possible to construct a 2*2 table |
| (Bailey et al., 2011b) | TP and FP are given, but FP and TN are not extractable |
| (Bailey et al., 2014) | Simulated threshold alert rates were performed |
| (Boom et al., 2014) | Purpose of the study was to compare minimally invasive glucose monitoring (intervention) with frequent capillary measurements (control)  The provided data was not sufficient to construct a 2*2 table |
| (Bondia et al., 2008) | Obtained dataset was retrospectively trained by support vector machines |
| (Buckingham et al., 2011) | Just the true alert rate is given |
| (Burt et al., 2013) | Only number of detected hypoglycaemic events by POC and CGM is given |
| (Christiansen et al., 2013) | Authors report the percentage of correctly detected hypoglycaemic events, but no other data to construct 2*2 table |
| (Christiansen et al., 2019) | Data was retrospectively analysed |
| (Cichosz et al., 2014)  (Cichosz et al., 2015) | Data was retrospectively analysed |
| (Davey et al., 2010) | Total number of measurements and hypoglycaemic events are missing to calculate a 2*2 table |
| (Donsa et al., 2014) | The provided data was not sufficient to construct a 2*2 table, maybe duplicate data (Schaupp et al., 2015a) |
| (Eastman et al., 2002) | Sensitivity and specificity were retrospectively analysed via ROC analysis |
| (Edge et al., 2017) | Sensitivity is given, but not enough information to construct 2x2 table |
| (Facchinetti et al., 2011) | Retrospective analysis |
| (Farhy et al., 2005) | Continuous Glucose Error Grid during hypoglycaemica and total number of hypoglycaemic events is given, but the provided data was not sufficient to construct a 2*2 table |
| (Francescato et al., 2012) | FN and FP and number of measurements are given, but information is not sufficient to calculate 2*2 table |
| (Freckmann et al., 2019) | 2x2 table not constructable, one reference standard measurement was paired more than one time with an index test measurement |
| (Gandrud et al., 2004) | Not enough data to construct 2x2 table, no TNs as reference measurements were only done after hypoglycaemia |
| (Gandrud et al., 2007) | Not enough data to construct 2x2 table and reference standard was not available for all of the events |
| (Garg et al., 2006) | No information about FP TN, retrospective evaluation of the hypoglycemia warning system |
| (Garg and Jovanovic, 2006) | Sensitivity, specificity and PPV is given, but not enough data (e.g. hypoglycaemic events) to calculate 2*2 table |
| (Garg et al., 2009) | Number of hypoglycaemic events for CGM and SMBG is given, but not enough data to construct 2x2 table |
| (Gomez et al., 2013)  (Gomez et al., 2015) | Only number of detected events of POC and CGM is given, not enough data to construct 2*2 table |
| (Gonder-Frederick et al., 2017a) | CGM was reference standard |
| (Gonder-Frederick et al., 2017b) | Overall (hypoglycaemia and hyperglycaemia) detection rate is given, no specificity of hypoglycaemia detection, therefore data was not sufficient to construct a 2*2 table |
| (Hansen et al., 2018) | Only FN and TP is given |
| (Heckermann et al., 2010a)  (Heckermann et al., 2010b) | 2*2 table of most likely the same study does not match  Written to author, but did not receive data |
| (Hermanns et al., 2007) | Study is about detection of diabetic patients with hypoglycaemia problems via blinded CGM data, no reference standard was available |
| (Hermanns et al., 2014) | The provided data was not sufficient to construct a 2*2 table |
| (Hoi-Hansen et al., 2005) | Data was recalibrated, author worked retrospectively with datasets |
| (Iaione and Marques, 2005) | The EEG signals were used to train and test the detection methodology retrospectively |
| (Jensen et al., 2013) | Features in datasets were retrospectively analysed to detect hypoglycaemia |
| (Kamath et al., 2010) | True alert rate, missed alert rate is given, but total number of measurements is missing to calculate 2*2 table |
| (Keenan et al., 2010) | The data set was analysed retrospectively |
| (Keenan et al., 2012) | Sensor data was retrospectively processed, real time perspective is just simulated |
| (Kosiborod et al., 2013) | Numbers given in the table about hypoglycaemia detection do not match numbers given in the text |
| (Kropff et al., 2015) | Just number of hypoglycaemic events and false positive alarms are given, not enough data to construct 2*2 table  Authors indicate that the study was not powered for indicating differences in hypoglycaemia |
| (Leal et al., 2010) | Retrospective hypoglycaemia detection based on training on parts of the obtained data |
| (Los et al., 2016)  (Los et al., 2017) | Reference test was CGM |
| (Mahmoudi et al., 2014b)  (Mahmoudi et al., 2014a) | Retrospective analysis of pre-existing datasets, calibration algorithm was applied retrospectively, dataset was provided by Novo Nordisk |
| (Mahmoudi et al., 2014c) | Retrospective analysis of pre-existing datasets |
| (Maia and Araujo, 2005)  (Maia and Araujo, 2006)  (Maia and Araujo, 2007) | Retrospective analysis of dataset |
| (Testa et al., 2018) | Not enough data to calculate 2x2 table |
| (Massa et al., 2018) | Number of correctly detected hypoglycaemic events and total number of hypogylcaemic events is reported, but no information is given about false positive or true negative events  The provided data was not sufficient to construct a 2*2 table |
| (McGarraugh and Bergenstal, 2009) | Clinic study: retrospective analysis of (Weinstein et al., 2007)  Home study: only data about number of events, which were proceed by capillary measurement, but no information about true detection, false alarms  The provided data was not sufficient to construct a 2*2 table |
| (McGarraugh, 2010) | Retrospective analysis of (McGarraugh and Bergenstal, 2009) |
| (McGarraugh et al., 2011) | total number of measurements in 2x2 table does not match the total number of measurements of the study  The provided data was not sufficient to construct a 2*2 table  Written to author, but did not receive data |
| (Meschi et al., 2010) | Only the number of patients with nocturnal hypoglycaemia is given, no comparision to reference standard is presented |
| (Norgaard et al., 2015) | Correctly detected events, not detected events, alters verified by events and false alters are given in percentage, the total number of events or measurements and the TN are not available  The provided data was not sufficient to construct a 2*2 table |
| (Obermaier et al., 2013)  (Zschornack et al., 2013) | Authors used a simulated alarm |
| (Peyser et al., 2013) | True alert rate is given, but not enough information to reconstruct 2*2 table |
| (Peyser et al., 2015) | Retrospective analysis |
| (Pieri et al., 2016) | Article consist of two experiments:  the first one compared CGM to capillary blood and no hypoglycaemic values were recorded  in the second CGM was recorded during scuba dive, hypoglycaemic values were recorded, but no reference standard was used |
| (Pitzer et al., 2001) | Retrospective analysis of the data of four trials |
| (Skladnev et al., 2010) | 2x2 table for actual values not constructable |
| (Tansey, 2005)  (Tsalikian, 2004a)  (Tsalikian, 2004b)  (Tsalikian et al., 2004)  Diabetes Research in Children Network (DirecNet) | Retrospective calibration no data about real-time sensitivity available |
| (Sachedina and Pickup, 2003) | Authors compare duration of hypoglycaemia between CGSM and reference standard and provide number of patients experiencing hypoglycaemia, but not enough data given to reconstruct 2*2 table |
| (Schaupp et al., 2015b) | Retrospective analysis of the data as CGM system was calibrated retrospectively, not all data was analysed as there was a data selection process to obtain datasets to analyse |
| (Schechter et al., 2012) | Only when a hypoglycaemic event occurred capillary glucose measurement were performed, otherwise CGM were used as reference standard |
| (Snogdal et al., 2012) | Just an approximate of sensitivity and specificity is given  The provided data was not sufficient to construct a 2*2 table |
| (Wadwa et al., 2018) | calculation of TP differs whether it is calculated via true alert rate an correct detection rate, reason might be the time interval of 15 minutes  The provided data was not sufficient to construct a 2*2 table  Written to author, but did not receive data |
| (Wang et al., 2015) | Sensitivity and specificity of nocturnal hypoglycaemia detection is given, number of hyoglycaemic alarams is given, but not number of hypoglycaemic events  The provided data was not sufficient to construct a 2*2 table |
| (Weinstein and McGarraugh, 2004) | Duplicate data (Weinstein et al., 2007) |
| (Weinstein et al., 2007) | Data was postprocessed to assess the accuracy of threshold alarms |
| (Weinzimer et al., 2005) | the total number do not match total number of paired measurements or any other known number  Written to author, but did not receive data |
| (Wentholt et al., 2005) | total number of hypoglycaemic events or total number of alarms at a specific threshold is missing  The provided data was not sufficient to construct a 2*2 table |
| (Wentholt et al., 2006) | Retrospective analysis of (Wentholt et al., 2005) |
| (Zaharieva et al., 2019) | The given numbers do not sum up to the total number of paired readings.  The provided data was not sufficient to construct a 2*2 table.  The analysis might be retrospective.  Written to author, but did not receive data |

**References:**

ADOLFSSON, P., NILSSON, S. & LINDBLAD, B. 2011. Continuous glucose monitoring system during physical exercise in adolescents with type 1 diabetes. *Acta Paediatrica,* 100**,** 1603-1609.

ALVA, S., BAILEY, T.et al. 2020. Accuracy of a 14-Day Factory-Calibrated Continuous Glucose Monitoring System With Advanced Algorithm in Pediatric and Adult Population With Diabetes. J Diabetes Sci Technol: 1932296820958754.

BAILEY, T., ZISSER, H., NAKAMURA, K., CHANG, A. & LILJENQUIST, D. 2011a. Continuous glucose error grid analysis (CG-EGA) of the prototype 4th generation dexcom continuous glucose monitoring system and frequently sampled self monitoring blood glucose (SMBG) data compare favorably to a laboratory standard. *Diabetes,* 60**,** A242.

BAILEY, T. S., AHMANN, A., BRAZG, R., CHRISTIANSEN, M., GARG, S., WATKINS, E., WELSH, J. B. & LEE, S. W. 2014. Accuracy and acceptability of the 6-day Enlite continuous subcutaneous glucose sensor. *Diabetes technology & therapeutics* [Online]. Available: <http://cochranelibrary-wiley.com/o/cochrane/clcentral/articles/000/CN-01117000/frame.html>.

BAILEY, T. S., ZISSER, H., NAKAMURA, K., CHANG, A. & LILJENQUIST, D. 2011b. Prototype 4th generation of dexcom continuous glucose monitoring system with improved home alert rates. *Diabetes,* 60**,** A238-A239.

BONDIA, J., TARIN, C., GARCIA-GABIN, W., ESTEVE, E., FERNANDEZ-REAL, J. M., RICART, W. & VEHI, J. 2008. Using support vector machines to detect therapeutically incorrect measurements by the MiniMed CGMS. *J Diabetes Sci Technol,* 2**,** 622-9.

BOOM, D. T., SECHTERBERGER, M. K., RIJKENBERG, S., KREDER, S., BOSMAN, R. J., WESTER, J. P., VAN STIJN, I., DEVRIES, J. H. & VAN DER VOORT, P. H. 2014. Insulin treatment guided by subcutaneous continuous glucose monitoring compared to frequent point-of-care measurement in critically ill patients: a randomized controlled trial. *Crit Care,* 18**,** 453.

BUCKINGHAM, B., LILJENQUIST, D., NAKAMURA, K., REALSEN, J., BENASSI, K. & CHASE, P. 2011. Effectiveness and safety study of the prototype 4th generation dexcom seven day continuous glucose monitoring system in youths with type 1 diabetes mellitus. *Diabetes,* 60**,** A335.

BURT, M. G., ROBERTS, G. W., AGUILAR-LOZA, N. R. & STRANKS, S. N. 2013. Brief Report: Comparison of Continuous Glucose Monitoring and Finger-Prick Blood Glucose Levels in Hospitalized Patients Administered Basal-Bolus Insulin. *Diabetes technology & therapeutics,* 15**,** 241-245.

CHRISTIANSEN, M., BAILEY, T., WATKINS, E., LILJENQUIST, D., PRICE, D., NAKAMURA, K., BOOCK, R. & PEYSER, T. 2013. A new-generation continuous glucose monitoring system: Improved accuracy and reliability compared with a previous-generation system. *Diabetes Technology and Therapeutics,* 15**,** 881-888.

CHRISTIANSEN, M. P., KLAFF, L. J., BAILEY, T. S., BRAZG, R., CARLSON, G. & TWEDEN, K. S. 2019. A Prospective Multicenter Evaluation of the Accuracy and Safety of an Implanted Continuous Glucose Sensor: The PRECISION Study. *Diabetes Technol Ther,* 21**,** 231-237.

CICHOSZ, S. L., FRYSTYK, J., HEJLESEN, O. K., TARNOW, L. & FLEISCHER, J. 2014. A novel algorithm for prediction and detection of hypoglycemia based on continuous glucose monitoring and heart rate variability in patients with type 1 diabetes. *Journal of Diabetes Science and Technology,* 8**,** 731-737.

CICHOSZ, S. L., FRYSTYK, J., TARNOW, L. & FLEISCHER, J. 2015. Combining information of autonomic odulation and CGM measurements nables prediction and improves etection of spontaneous hypoglycemic vents. *Journal of Diabetes Science and Technology,* 9**,** 132-137.

DAVEY, R. J., JONES, T. W. & FOURNIER, P. A. 2010. Effect of short-term use of a continuous glucose monitoring system with a real-time glucose display and a low glucose alarm on incidence and duration of hypoglycemia in a home setting in type 1 diabetes mellitus. *Journal of Diabetes Science and Technology* [Online]. Available: <http://cochranelibrary-wiley.com/o/cochrane/clcentral/articles/379/CN-00779379/frame.html>.

DONSA, K., NEUBAUER, K. M., MADER, J. K., HOLL, B., SPAT, S., TSCHAPELLER, B., BECK, P., PLANK, J., PIEBER, T. R. & SCHAUPP, L. 2014. Are we missing something? continuous glucose monitoring compared with poct among hospitalized type 2 diabetes patients on basal-bolus insulin therapy. *Diabetes Technology and Therapeutics,* 16**,** A7.

EASTMAN, R. C., CHASE, H. P., BUCKINGHAM, B., HATHOUT, E. H., LEPTIEN, A., VAN WYHE, M., DAVIS, T., WEI, C., TAMADA, J. A. & PITZER, K. R. 2002. Use of the GlucoWatch (R) biographer in children and adolescents with diabetes. *Diabetes,* 51**,** A121-A121.

EDGE, J., ACERINI, C., CAMPBELL, F., HAMILTON-SHIELD, J., MOUDIOTIS, C., RAHMAN, S., RANDELL, T., SMITH, A. & TREVELYAN, N. 2017. An alternative sensor-based method for glucose monitoring in children and young people with diabetes. *Archives of Disease in Childhood,* 102**,** 543-549.

FACCHINETTI, A., SPARACINO, G., CALORE, F. & COBELLI, C. 2011. On-line CGM denoising improves hypo/hyperalert generation. *Diabetes Technology and Therapeutics,* 13**,** 223.

FARHY, L. S., KOVATCHEV, B. P., GONDER-FREDERICK, L. A., COX, D. J., ANDERSON, S. M. & CLARKE, W. L. 2005. Accuracy of FreeStyle Navigator (TM) and MiniMed CGMS (R) during euglycemia and induced Hypoglycemia. *Diabetes,* 54**,** A97-A97.

FRANCESCATO, M. P., GEAT, M., STEL, G. & CAUCI, S. 2012. Accuracy of a portable glucose meter and of a Continuous Glucose Monitoring device used at home by patients with type 1 diabetes. *Clinica Chimica Acta,* 413**,** 312-318.

FRECKMANN, G., LINK, M., KAMECKE, U., HAUG, C., BAUMGARTNER, B. & WEITGASSER, R. 2019. Performance and Usability of Three Systems for Continuous Glucose Monitoring in Direct Comparison. *Journal of Diabetes Science and Technology.*

GANDRUD, L. M., PAGUNTALAN, H. U., VAN WYHE, M. M., KUNSELMAN, B. L., LEPTIEN, A. D., WILSON, D. M., EASTMAN, R. C. & BUCKINGHAM, B. A. 2004. Use of the Cygnus GlucoWatch biographer at a diabetes camp. *Pediatrics,* 113**,** 108-11.

GANDRUD, L. M., XING, D., KOLLMAN, C., BLOCK, J. M., KUNSELMAN, B., WILSON, D. M. & BUCKINGHAM, B. A. 2007. The Medtronic MiniMed gold continuous glucose monitoring system: An effective means to discover hypo- and hyperglycemia in children under 7 years of age. *Diabetes Technology and Therapeutics,* 9**,** 307-316.

GARG, S. & JOVANOVIC, L. 2006. Relationship of fasting and hourly blood glucose levels to HbA<inf>1c</inf> values: Safety, accuracy, and improvements in glucose profiles obtained using a 7-day continuous glucose sensor. *Diabetes Care,* 29**,** 2644-2649.

GARG, S., ZISSER, H., SCHWARTZ, S., BAILEY, T., KAPLAN, R., ELLIS, S. & JOVANOVIC, L. 2006. Improvement in glycemic excursions with a transcutaneous, real-time continuous glucose sensor: A randomized controlled trial. *Diabetes Care,* 29**,** 44-50.

GARG, S. K., VOELMLE, M. K. & GOTTLIEB, P. 2009. Feasibility of 10-Day Use of a Continuous Glucose-Monitoring System in Adults With Type 1 Diabetes. *Diabetes Care,* 32**,** 436-438.

GOMEZ, A. M., UMPIERREZ, G. E., ASCHNER, P., HERRERA, F. & MUNOZ, O. 2013. Comparison of inpatient glycaemic control by continuous glucose monitoring (CGM) and capillary point-of-care (POC) testing in general medicine patients with type 2 diabetes. *Diabetologia,* 56**,** S30-S30.

GOMEZ, A. M., UMPIERREZ, G. E., MUNOZ, O. M., HERRERA, F., RUBIO, C., ASCHNER, P. & BUENDIA, R. 2015. Continuous Glucose Monitoring Versus Capillary Point-of-Care Testing for Inpatient Glycemic Control in Type 2 Diabetes Patients Hospitalized in the General Ward and Treated With a Basal Bolus Insulin Regimen. *J Diabetes Sci Technol,* 10**,** 325-9.

GONDER-FREDERICK, L. A., GRABMAN, J. H. & SHEPARD, J. A. 2017a. Diabetes Alert Dogs (DADs): An assessment of accuracy and implications. *Diabetes Research and Clinical Practice,* 134**,** 121-130.

GONDER-FREDERICK, L. A., GRABMAN, J. H., SHEPARD, J. A., TRIPATHI, A. V., DUCAR, D. M. & MCELGUNN, Z. R. 2017b. Variability of Diabetes Alert Dog Accuracy in a Real-World Setting. *J Diabetes Sci Technol,* 11**,** 714-719.

HANSEN, E. A., KLEE, P., DIRLEWANGER, M., BOUTHORS, T., ELOWE-GRUAU, E., STOPPA-VAUCHER, S., PHAN-HUG, F., ANTONIOU, M. C., PASQUIER, J., DWYER, A. A., PITTELOUD, N. & HAUSCHILD, M. 2018. Accuracy, satisfaction and usability of a flash glucose monitoring system among children and adolescents with type 1 diabetes attending a summer camp. *Pediatric Diabetes,* 19**,** 1276-1284.

HECKERMANN, S., NOSEK, L., ZIJLSTRA, E. & HEISE, T. 2010a. Accuracy of a continuous glucose monitoring system (CGMS): still room for improvement. *Diabetologia,* 53**,** S26-S27.

HECKERMANN, S., NOSEK, L., ZIJLSTRA, E. & HEISE, T. 2010b. Accuracy of a Continuous Glucose Monitoring System (CGMS): Still Room for Improvement. *Diabetes,* 59**,** A131-A132.

HERMANNS, N., KULZER, B., KUBIAK, T., KRICHBAUM, M. & HAAK, T. 2007. The effect of an education programme (HyPOS) to treat hypoglycaemia problems in patients with type 1 diabetes. *Diabetes/Metabolism Research and Reviews* [Online]. Available: <http://cochranelibrary-wiley.com/o/cochrane/clcentral/articles/641/CN-00617641/frame.html>.

HERMANNS, N., SCHUMANN, B., KULZER, B. & HAAK, T. 2014. The impact of continuous glucose monitoring on low interstitial glucose values and low blood glucose values assessed by point-of-care blood glucose meters: results of a crossover trial. *J Diabetes Sci Technol,* 8**,** 516-22.

HOI-HANSEN, T., PEDERSEN-BJERGAARD, U. & THORSTEINSSON, B. 2005. Reproducibility and reliability of hypoglycaemic episodes recorded with Continuous Glucose Monitoring System (CGMS) in daily life. *Diabet Med,* 22**,** 858-62.

IAIONE, F. & MARQUES, J. L. B. 2005. Methodology for hypoglycaemia detection based on the processing, analysis and classification of the electroencephalogram. *Medical & biological engineering & computing,* 43**,** 501-507.

JENSEN, M. H., CHRISTENSEN, T. F., TARNOW, L., JOHANSEN, M. D. & HEJLESEN, O. K. 2013. Characterization of hypoglycemia by processing subcutaneous sensor and insulin data. *Journal of Diabetes Science and Technology,* 7**,** A64.

KAMATH, A., MAHALINGAM, A. & BRAUKER, J. 2010. Methods of evaluating the utility of continuous glucose monitor alerts. *Journal of Diabetes Science and Technology,* 4**,** 57-66.

KEENAN, D. B., CARTAYA, R. & MASTROTOTARO, J. J. 2010. Accuracy of a new real-time continuous glucose monitoring algorithm. *Journal of Diabetes Science and Technology,* 4**,** 111-118.

KEENAN, D. B., MASTROTOTARO, J. J., ZISSER, H., COOPER, K. A., RAGHAVENDHAR, G., LEE, S. W., YUSI, J., BAILEY, T. S., BRAZG, R. L. & SHAH, R. V. 2012. Accuracy of the enlite 6-day glucose sensor with Guardian and Veo calibration algorithms. *Diabetes Technology and Therapeutics,* 14**,** 225-231.

KOSIBOROD, M., GOTTLIEB, R., SEKELLA, J., PETERMAN, D., GRODZINSKY, A., KENNEDY, P. & BORKON, M. 2013. Performance of the Medtronic Sentrino continuous glucose management system in the cardiac ICU. *Critical Care,* 17**,** S172.

KROPFF, J., BRUTTOMESSO, D., DOLL, W., FARRET, A., GALASSO, S., LUIJF, Y. M., MADER, J. K., PLACE, J., BOSCARI, F., PIEBER, T. R., RENARD, E. & DEVRIES, J. H. 2015. Accuracy of two continuous glucose monitoring systems: A head-to-head comparison under clinical research centre and daily life conditions. *Diabetes, Obesity and Metabolism,* 17**,** 343-349.

LEAL, Y., GARCIA-GABIN, W., BONDIA, J., ESTEVE, E., RICART, W., FERNANDEZ-REAL, J. M. & VEHI, J. 2010. Real-time glucose estimation algorithm for continuous glucose monitoring using autoregressive models. *Journal of Diabetes Science and Technology,* 4**,** 391-403.

LOS, E. A., GUTTMANN-BAUMAN, I., RAMSEY, K. & AH-MANN, A. J. 2016. Reliability of trained dogs to detect hypoglycemia in type 1 diabetes. *Diabetes,* 65**,** A20.

LOS, E. A., RAMSEY, K. L., GUTTMANN-BAUMAN, I. & AHMANN, A. J. 2017. Reliability of Trained Dogs to Alert to Hypoglycemia in Patients with Type 1 Diabetes. *Journal of Diabetes Science and Technology,* 11**,** 506-512.

MAHMOUDI, Z., HASSELSTROM JENSEN, M., DENCKER JOHANSEN, M., CHRISTENSEN, T. F., TARNOW, L., CHRISTIANSEN, J. S. & HEJLESEN, O. K. 2014a. Performance assessment of a continuous glucose monitoring calibration algorithm in hypoglycemia. *Diabetes Technology and Therapeutics,* 16**,** A75.

MAHMOUDI, Z., JENSEN, M. H., DENCKER JOHANSEN, M., CHRISTENSEN, T. F., TARNOW, L., CHRISTIANSEN, J. S. & HEJLESEN, O. 2014b. Accuracy evaluation of a new real-time continuous glucose monitoring algorithm in hypoglycemia. *Diabetes Technology and Therapeutics,* 16**,** 667-678.

MAHMOUDI, Z., JOHANSEN, M. D., CHRISTIANSEN, J. S. & HEJLESEN, O. 2014c. Comparison between one-point calibration and two-point calibration approaches in a continuous glucose monitoring algorithm. *Journal of Diabetes Science and Technology,* 8**,** 709-719.

MAIA, F. F. & ARAUJO, L. R. 2005. Efficacy of continuous glucose monitoring system to detect unrecognized hypoglycemia in children and adolescents with type 1 diabetes. *Arquivos brasileiros de endocrinologia e metabologia,* 49**,** 569-574.

MAIA, F. F. R. & ARAUJO, L. R. 2006. Metabolic / glycemic control accuracy, effect and complications of the continuous glucose monitoring system in diabetic patients. *Revista da Associacao Medica Brasileira,* 52**,** 395-400.

MAIA, F. F. R. & ARAUJO, L. R. 2007. Efficacy of continuous glucose monitoring system (CGMS) to detect postprandial hyperglycemia and unrecognized hypoglycemia in type 1 diabetic patients. *Diabetes Research and Clinical Practice,* 75**,** 30-34.

MASSA, G. G., GYS, I., OP 'T EYNDT, A., BEVILACQUA, E., WIJNANDS, A., DECLERCQ, P. & ZEEVAERT, R. 2018. Evaluation of the FreeStyle® Libre Flash Glucose Monitoring System in Children and Adolescents with Type 1 Diabetes. *Hormone Research in Paediatrics,* 89**,** 189-199.

MCGARRAUGH, G. 2010. Alarm characterization for a continuous glucose monitor that replaces traditional blood glucose monitoring. *Journal of Diabetes Science and Technology,* 4**,** 49-56.

MCGARRAUGH, G. & BERGENSTAL, R. 2009. Detection of hypoglycemia with continuous interstitial and traditional blood glucose monitoring using the FreeStyle navigator continuous glucose monitoring system. *Diabetes Technology and Therapeutics,* 11**,** 145-150.

MCGARRAUGH, G., BRAZG, R. & WEINSTEIN, R. 2011. FreeStyle navigator continuous glucose monitoring system with TRUstart algorithm, a 1-hour warm-up time. *Journal of Diabetes Science and Technology,* 5**,** 99-106.

MESCHI, F., BONFANTI, R., RIGAMONTI, A., FRONTINO, G., BATTAGLINO, R., VISCARDI, M., POSCIA, A. & CHIUMELLO, G. 2010. Patients' evaluation of nocturnal hypoglycaemia with GlucoDay continuous glucose monitoring in paediatric patients. *Acta Diabetologica,* 47**,** 295-300.

NORGAARD, K., SHIN, J., WELSH, J. B. & GJESSING, H. 2015. Performance and acceptability of a combined device for insulin infusion and glucose sensing in the home setting. *Journal of Diabetes Science and Technology,* 9**,** 215-220.

OBERMAIER, K., SCHOEMAKER, M., JAGER, J., SCHMELZEISEN-REDEKER, G., FRECKMANN, G. & ZSCHORNACK, E. 2013. Performance of a novel sensor during induced glucose swings. *Diabetologia,* 56**,** S435.

PEYSER, T., BOHNETT, L. & NAKAMURA, K. 2013. Improved hypoglycemic accuracy, alerts and detection with the G4 platinum cgm system. *Diabetes,* 62**,** A99-A100.

PEYSER, T. A., NAKAMURA, K., PRICE, D., BOHNETT, L. C., HIRSCH, I. B. & BALO, A. 2015. Hypoglycemic Accuracy and Improved Low Glucose Alerts of the Latest Dexcom G4 Platinum Continuous Glucose Monitoring System. *Diabetes Technology and Therapeutics,* 17**,** 548-554.

PIERI, M., CIALONI, D. & MARRONI, A. 2016. Continuous real-time monitoring and recording of glycemia during scuba diving: Pilot study. *Undersea and Hyperbaric Medicine,* 43**,** 265-272.

PITZER, K. R., DESAI, S., DUNN, T., EDELMAN, S., JAYALAKSHMI, Y., KENNEDY, J., TAMADA, J. A. & POTTS, R. O. 2001. Detection of hypoglycemia with the GlucoWatch biographer. *Diabetes Care,* 24**,** 881-5.

SACHEDINA, N. & PICKUP, J. C. 2003. Performance assessment of the Medtronic-MiniMed Continuous Glucose Monitoring System and its use for measurement of glycaemic control in Type 1 diabetic subjects. *Diabetic Medicine,* 20**,** 1012-1015.

SCHAUPP, L., DONSA, K., NEUBAUER, K. M., MADER, J. K., ABERER, F., HOLL, B., SPAT, S., AUGUSTIN, T., BECK, P., PIEBER, T. R. & PLANK, J. 2015a. Taking a Closer Look--Continuous Glucose Monitoring in Non-Critically Ill Hospitalized Patients with Type 2 Diabetes Mellitus Under Basal-Bolus Insulin Therapy. *Diabetes Technol Ther,* 17**,** 611-8.

SCHAUPP, L., DONSA, K., NEUBAUER, K. M., MADER, J. K., ABERER, F., HOLL, B., SPAT, S., AUGUSTIN, T., BECK, P., PIEBER, T. R. & PLANK, J. 2015b. Taking a Closer Look - Continuous Glucose Monitoring in Non-Critically Ill Hospitalized Patients with Type 2 Diabetes Mellitus Under Basal-Bolus Insulin Therapy. *Diabetes Technology and Therapeutics,* 17**,** 611-618.

SCHECHTER, A., EYAL, O., ZUCKERMAN-LEVIN, N., AMIHAI-BEN-YAACOV, V., WEINTROB, N. & SHEHADEH, N. 2012. A prototype of a new noninvasive device to detect nocturnal hypoglycemia in adolescents with type 1 diabetes-A pilot study. *Diabetes Technology and Therapeutics,* 14**,** 683-689.

SKLADNEV, V. N., GHEVONDIAN, N., TARNAVSKII, S., PARAMALINGAM, N. & JONES, T. W. 2010. Clinical evaluation of a noninvasive alarm system for nocturnal hypoglycemia. *J Diabetes Sci Technol,* 4**,** 67-74.

SNOGDAL, L. S., FOLKESTAD, L., ELSBORG, R., REMVIG, L. S., BECK-NIELSEN, H., THORSTEINSSON, B., JENNUM, P., GJERSTAD, M. & JUHL, C. B. 2012. Detection of hypoglycemia associated EEG changes during sleep in type 1 diabetes mellitus. *Diabetes Research and Clinical Practice,* 98**,** 91-97.

TANSEY, M. J. 2005. Accuracy of the modified continuous glucose monitoring system (CGMS®) sensor in an outpatient setting: Results from a diabetes research in children network (DirecNet) study. *Diabetes Technology and Therapeutics,* 7**,** 109-114.

TESTA, M. A., SALDIVAR-SALAZAR, S., SU, M., HAYES, J. F. & SIMONSON, D. C. 2018. Continuous glucose monitoring (CGM) provides enhanced sensitivity compared with self-monitored blood glucose (SMBG) for detecting hypoglycemia during T2D clinical trials. *Diabetes,* 67 (Supplement 1)**,** A244.

TSALIKIAN, E. 2004a. Accuracy of the GlucoWatch G2 Biographer and the Continuous Glucose Monitoring System during Hypoglycemia: Experience of the Diabetes Research in Children Network. *Diabetes Care,* 27**,** 722-726.

TSALIKIAN, E. 2004b. GlucoWatch® G2™ biographer alarm reliability during hypoglycemia in children. *Diabetes Technology and Therapeutics,* 6**,** 559-566.

TSALIKIAN, E., BECK, R. W., TAMBORLANE, W. V., CHASE, P., BUCKINGHAM, B. A., WEINZIMER, S. A., MAURAS, N., RUEDY, K. J., KOLLMAN, C., XING, D. Y., FIALLO-SCHARER, R., FISHER, J. H., TANSEY, M. J., LARSON, L. F., WYSOCKI, T., GAGNON, K. M., TODD, P., WILSON, D. M., BLOCK, J. M., KUNSELMAN, E. L., DOYLE, E. A., MOKE, P. S., LABASTIE, L. M., BECKER, D. M., COX, C., RYAN, C. M., WHITE, N. H., WHITE, P. C., STEFFES, M. W., BUCKSA, J. M., NOWICKI, M. L., GRAVE, G. D., LINDER, B., WINER, K. K. & DIRECNET STUDY, G. 2004. Accuracy of the GlucoWatch G2 Biographer and the continuous glucose monitoring system during hypoglycemia - Experience of the Diabetes Research in Children Network. *Diabetes Care,* 27**,** 722-726.

WADWA, R. P., LAFFEL, L. M., SHAH, V. N. & GARG, S. K. 2018. Accuracy of a Factory-Calibrated, Real-Time Continuous Glucose Monitoring System During 10 Days of Use in Youth and Adults with Diabetes. *Diabetes Technol Ther*.

WANG, X., IOACARA, S. & DEHENNIS, A. 2015. Long-Term Home Study on Nocturnal Hypoglycemic Alarms Using a New Fully Implantable Continuous Glucose Monitoring System in Type 1 Diabetes. *Diabetes Technology and Therapeutics,* 17**,** 780-786.

WEINSTEIN, R. & MCGARRAUGH, G. V. 2004. Accuracy evaluation of the FreeStyle Navigator (TM) Continuous Glucose Monitor - Detection of hypoglycemia. *Diabetes,* 53**,** A107-A107.

WEINSTEIN, R. L., BUGLER, J. R., SCHWARTZ, S. L., PEYSER, T. A., BRAZG, R. L. & MCGARRAUGH, G. V. 2007. Accuracy of the 5-day freestyle navigator continuous glucose monitoring system - Comparison with frequent laboratory reference measurements. *Diabetes Care,* 30**,** 1125-1130.

WEINZIMER, S. A., BECK, R. W., CHASE, H. P., FOX, L. A., BUCKINGHAM, B. A., TAMBORLANE, W. V., KOLLMAN, C., COFFEY, J., XING, D., RUEDY, K. J. & DIABETES RESEARCH IN CHILDREN NETWORK STUDY, G. 2005. Accuracy of newer-generation home blood glucose meters in a Diabetes Research in Children Network (DirecNet) inpatient exercise study. *Diabetes Technol Ther,* 7**,** 675-80; discussion 681-3.

WENTHOLT, I. M., HOEKSTRA, J. B. & DEVRIES, J. H. 2006. A critical appraisal of the continuous glucose-error grid analysis. *Diabetes Care,* 29**,** 1805-1811.

WENTHOLT, I. M., VOLLEBREGT, M. A., HART, A. A., HOEKSTRA, J. B. & DEVRIES, J. H. 2005. Comparison of a needle-type and a microdialysis continuous glucose monitor in type 1 diabetic patients. *Diabetes Care,* 28**,** 2871-2876.

ZAHARIEVA, D. P., TURKSOY, K., MCGAUGH, S. M., POONI, R., VIENNEAU, T., LY, T. & RIDDELL, M. C. 2019. Lag Time Remains with Newer Real-Time Continuous Glucose Monitoring Technology During Aerobic Exercise in Adults Living with Type 1 Diabetes. *Diabetes Technol Ther,* 21**,** 313-321.

ZSCHORNACK, E., SCHMID, C., PLEUS, S., LINK, M., KLOTZER, H. M., OBERMAIER, K., SCHOEMAKER, M., STRASSER, M., FRISCH, G., SCHMELZEISEN-REDEKER, G., HAUG, C. & FRECKMANN, G. 2013. Evaluation of the performance of a novel system for continuous glucose monitoring. *J Diabetes Sci Technol,* 7**,** 815-23.

**Supplement 3**

**Part one: Reviewer and Study information**

Reviewer Name:

Title:

Source (journal, year, volume, pages):

Authors:

Type of report: full paper/ conference abstract/ conference presentation/ other

Please indicate if other:

**Part two: Information on the study:**

| **2.1 Characteristics of the trial** |  |
| --- | --- |
| Country of conduction: |  |
| Country of authors: |  |
| Funders: | manufacturer/ other/ not given  If other please indicate: |
| Year of conduction: |  |
| Setting:  (please indicate all study settings) | Setting 1: hospital/ research centre/ home/other  If other please indicate:  Setting 2: hospital/ research centre/ home |
| Was the trial a multi-centre study? | yes/ no/ unclear  How many centres were there? |
| Was an insulin challenge administered? | yes/ no/ unclear |

| **2.2 Characteristics of the participants** |  |
| --- | --- |
| Inclusion criteria: | |
| Exclusion criteria: | |
| Participants characteristics: (only mean value, no SD or range, has to be extracted) | |
| Total number of included participants: |  |
| Total number of analysed participants: |  |
| Reasons for drop out: |  |

| **2.3 Index test(s)**  (please indicate all examined index tests) |  |
| --- | --- |
|  | |
| **Index Test 1** |  |
| Number of paired measurements: |  |
| Technique: | minimally invasive/ non invasive |
| Exact name of device: |  |
| Insertion/Measurement site:  (if more than one please indicate all) |  |
| Person inserting/applying the device: | participant or parent/ healthcare professional/ other/ information not given  If other please indicate |
| Person executing calibration | participant or parent/ healthcare professional/ other/ information not given  If other please indicate |
| In which time-interval had the index test to be positive? |  |
| Length of sensor wear: |  |
| At which time of the day was measured? | day and night/ only day/ only night/ not given |
|  | |
| Was a second index test/calibration algorithm used? | yes/ no |
| **If not please continue with reference test (2.4)** | |
| **Index Test 2:** |  |
| Number of paired measurements: |  |
| Technique | minimally invasive/ non invasive |
| Exact name of device: |  |
| Insertion/Measurement site:  (if more than one please indicate all) |  |
| Person inserting/applying the device: | participant or parent/ healthcare professional/ other/ information not given  If other please indicate |
| Person executing calibration | participant or parent/ healthcare professional/ other/ information not given  If other please indicate |
| In which time-interval had the index test to be positive? |  |
| Length of sensor wear: |  |
|  | |
| Was a third index test/calibration algorithm used? | yes/ no |
| **If not please continue with reference test (2.4)** |  |
| **Index Test 3:** |  |
| Number of paired measurements: |  |
| Technique | minimally invasive/ non invasive |
| Exact name of device: |  |
| Insertion/Measurement site:  (if more than one please indicate all) |  |
| Person inserting/applying the device: | participant or parent/ healthcare professional/ other/ information not given  If other please indicate |
| Person executing calibration | participant or parent/ healthcare professional/ other/ information not given  If other please indicate |
| In which time-interval had the index test to be positive? |  |
| Length of sensor wear: |  |

| **2.4 Reference test(s)**  (please indicate all used reference tests) |  |
| --- | --- |
|  | |
| **Reference Test 1** |  |
| Technique: | capillary blood/ venous blood/ arterial blood/ other |
| Exact name of device: |  |
| Timing of measurement/  Number of measurements |  |
| Time interval between index and reference test (and treatments  carried out in between) | yes/ no/ not given  if yes please indicate: |
|  | |
| Was a second reference test used? | yes/ no |
| **If not please continue with primary outcome (2.5)** |  |
| **Reference Test 2** |  |
| Technique: | capillary blood/ venous blood/ arterial blood/ other |
| Exact name of device: |  |
| Timing of measurement/  Number of measurements |  |
| Time interval between index and reference test (and treatments  carried out in between) | yes/ no/ not given  if yes please indicate: |
| Was a second reference test used? | yes/ no |
|  | |
| **If not please continue with primary outcome (2.5)** |  |
| **Reference Test 3** |  |
| Technique: | capillary blood/ venous blood/ arterial blood/ other |
| Exact name of device: |  |
| Timing of measurement/  Number of measurements |  |
| Time interval between index and reference test (and treatments  carried out in between) | yes/ no/ not given  if yes please indicate |

| **2.5 Primary outcome:**  **(Accuracy of hypoglycaemia detection)** | |
| --- | --- |
| **Threshold 1:** | |
| Threshold for index test:  Threshold for reference test: | |
| 2*2 table about hypoglycaemia detection  Place for calculation   \|  \| Reference test positive/ <threshold \| Reference test negative/ >threshold \| Total \| \| --- \| --- \| --- \| --- \| \| Index test positive/ <threshold \|  \|  \|  \| \| Index test negative/ >threshold \|  \|  \|  \| \| Total \|  \|  \|  \| | |
| Sensitivity: | % |
| Specificity: | % |
| Was additional relevant diagnostic accuracy data given?  (e.g. false positive rate, true detection rate, positive/negative predictive value): | yes/ no/ already given above at calculation  If yes please indicate with given value: |
| Number of uninterpretable/excluded measurements |  |
| Information about sensor age/time of day/ special circumstances? |  |
| On which measurement is the 2*2 table based? | all paired measurements/ measurements at a specific point in time/ number of nights/ other  unclear  If other/unclear please indicate: |
| Was the 2*2 table given by the authors? | yes/ no |
|  | |
| Was a second pre-specified threshold/calibration method/ comparison to different reference standard used? | yes/ no |
| **If not please continue with additional outcomes (2.6)** | |
| **Threshold 2:** | |
| Threshold for index test:  Threshold for reference test: | |
| 2*2 table about hypoglycaemia detection  Place for calculation   \|  \| Reference test positive/ <threshold \| Reference test negative/ >threshold \| Total \| \| --- \| --- \| --- \| --- \| \| Index test positive/ <threshold \|  \|  \|  \| \| Index test negative/ >threshold \|  \|  \|  \| \| Total \|  \|  \|  \| | |
| Sensitivity: | % |
| Specificity: | % |
| Was additional relevant diagnostic accuracy data given?  (e.g. false positive rate, true detection rate, positive/negative predictive value): | yes/ no/ already given above at calculation  If yes please indicate with given value: |
| On which measurement is the 2*2 table based? | all paired measurements/ measurements at a specific point in time/ number of nights/ other  unclear  If other/unclear please indicate: |
|  |  |
| Was a third pre-specified threshold/calibration method used? | yes/ no |
| **If not please continue with additional outcomes (2.6)** | |
| **Threshold 3:** |  |
|  | |
| Threshold for index test:  Threshold for reference test: | |
| 2*2 table about hypoglycaemia detection  Place for calculation   \|  \| Reference test positive/ <threshold \| Reference test negative/ >threshold \| Total \| \| --- \| --- \| --- \| --- \| \| Index test positive/ <threshold \|  \|  \|  \| \| Index test negative/ >threshold \|  \|  \|  \| \| Total \|  \|  \|  \| | |
| Sensitivity: | % |
| Specificity: | % |
| Was additional relevant diagnostic accuracy data given?  (e.g. false positive rate, true detection rate, positive/negative predictive value): | yes/ no/ already given above at calculation  If yes please indicate with given value: |
| Number of uninterpretable/excluded measurements |  |
| Information about sensor age/time of day/ special circumstances? |  |
| On which measurement is the 2*2 table based? | all paired measurements/ measurements at a specific point in time/ number of nights/ other  unclear  If other/unclear please indicate: |
|  | |

| **Additional outcomes:** |  |
| --- | --- |
| **2.6 Additional Accuracy Data at hypoglycaemia:** |  |
| Is additional accuracy data at hypoglycaemia given? | yes/ no |
| **If not please continue with additional general accuracy data (2.7)**  Otherwise please indicate all data which was given | |
| Threshold: |  |
| MARD (mean): |  |
| MARD (median): |  |
| (absolute) difference |  |
| Correlation Coefficient |  |
| Clarke Error Grid Analysis: | Zone A+B: |
| Consensus/Parkes Error Grid Analysis: | Zone A+B: |
| Continuous Error Grid Analysis (CG-EGA): | Zone A+B: |
| within±15 mg/dL for reference glucose values ≤75 mg/dL (ISO criteria) |  |
| Was there additional relevant data in the hypoglycaemic range? | yes/ no  If please indicate: |
|  |  |
| **2.7 Additional general accuracy data:** |  |
| Is additional accuracy data given?  (please only indicate data about the whole glycaemic range, no data of accuracy in the hyperglycaemic range necessary) | yes/ no |
| **If not please continue with additional outcome 2.8** | |
| **Index Test 1 compared to Reference Standard 1:** |  |
| Index Test 1: (name of device) |  |
| Reference Standard: | capillary blood/ venous blood/ arterial blood/ other |
| MARD (mean): |  |
| MARD (median): |  |
| (absolute) difference |  |
| Correlation Coefficient |  |
| Clarke Error Grid Analysis: | Zone A+B: |
| Consensus/Parkes Error Grid Analysis: | Zone A+B: |
| Continuous Error Grid Analysis (CG-EGA): | Zone A+B: |
| within±15%/dL for reference glucose values >100 mg/dL (ISO criteria) |  |
| within±20/20% criteria |  |
| Was there any additional general accuracy data?  (no extraction of accuracy in special circumstances (e.g. pressure, exercise, ROC, two sensors in one person) necessary) | yes/ no  If please indicate: |
| Was there any general accuracy data compared to another reference standard? | yes/ no |
| **If not please continue with Index Test 2** | |
| **Index Test 1 compared to Reference Standard 2:** | |
| Reference Standard: | capillary blood/ venous blood/ arterial blood/ other |
| MARD (mean): |  |
| MARD (median): | % |
| (absolute) difference |  |
| Correlation Coefficient |  |
| Clarke Error Grid Analysis: | Zone A+B: |
| Consensus/Parkes Error Grid Analysis: | Zone A+B: |
| Continuous Error Grid Analysis (CG-EGA): | Zone A+B: |
| within±15%/dL for reference glucose values >100 mg/dL (ISO criteria) |  |
| within±20/20% criteria |  |
| Was there any additional general accuracy data? | yes/ no |
|  | |
| **Index Test 2 compared to Reference Standard 1:** | |
| Was there any general accuracy data compared to another index test? | yes/ no |
| **If not please continue with additional outcome (2.8)** | |
| Reference Standard: | capillary blood/ venous blood/ arterial blood/ other |
| Index Test 2: (name of device) |  |
| MARD (mean): |  |
| MARD (median): |  |
| (absolute) difference |  |
| Correlation Coefficient |  |
| Clarke Error Grid Analysis: | Zone A+B: |
| Consensus/Parkes Error Grid Analysis: | Zone A+B: |
| Continuous Error Grid Analysis (CG-EGA): | Zone A+B: |
| within±15%/dL for reference glucose values >100 mg/dL (ISO criteria) |  |
| within±20/20% criteria |  |
| Was there any additional general accuracy data? | yes/ no  If please indicate: |

| **2.8. additional outcome** | |
| --- | --- |
| Information about sensor stability (removed sensors, calibration error?) | yes/ no  If please indicate: |
| Was there any information about side effects/ adverse events? | yes/ no  If please indicate: |

Do you have any additional comments? yes/ no

If yes please indicate:

Is further information required from the authors? yes/ no

If yes give details:

## Appendix 5 Review-tailored Quality Assessment of Diagnostic Accuracy Studies 2 (QUADAS-2) tool

**Domain 1: Patient Selection**

1.1 Was a consecutive or random sample of patients enrolled?

yes/ no/ unclear

Yes = it is explicitly stated in the study that enrolment was consecutive or random

No = sample was non-random or patients were not consecutively recruited, e.g. it was only recruited during day time

Unclear = information cannot be obtained from the paper

1.2 Was a case control design avoided?

yes/ no/ unclear

Yes = representative patients were recruited

No = concerns about representativeness, e.g. only patients with high risk of hypoglycaemia were included or hypoglycaemia was artificially achieved (insulin-challenge)

Unclear = information cannot be obtained from the paper

1.3. Did the study avoid inappropriate exclusions?

yes/ no/ unclear

Yes = exclusion criteria are clearly described and appropriate, e.g. pregnancy or children as exclusion criteria, insulin-treatment as inclusion criteria

No = exclusion criteria are clear but exclusion of patients who would be representative, e.g. patients with hypoglycaemia unawareness, history of severe hypoglycaemia

Unclear = information cannot be obtained from the paper

1.4 Risk of Bias: Could the selection of patients have introduced bias?

high/ low/ unclear

High = at least one question is “no”, indicating that there is concern

Low = all questions are answered “yes”

Unclear = answer to at least one question is “unclear” and none are answered “yes”

(relating to questions 1.1., 1.2., 1.3)

1.5 Concerns regarding applicability:

Are there concerns that the included patients do not match the review question?

high/ low/ unclear

High = included patients are inherently different from the patients who would be expected to use minimally or non-invasive glucose monitoring

Low = there are no such concerns

Unclear = patient characteristics are not sufficiently clearly explained to make a judgment on patient inclusion

Additional relevant information:

**Domain 2: Index Test**

2.1 Were the index test results interpreted without knowledge of the results of the reference standard?

yes/ no/ unclear

Yes = it is stated clearly that the individuals interpreting the index test did not know the results of the reference test

No = the results of the reference test were known by the individuals performing the index test

Unclear = information cannot be obtained from the paper

2.2 Was/were the threshold(s) was pre-specified?

yes/ no/ unclear

Yes = it is stated that the threshold(s) were pre-specified, the threshold is already clearly described in the method section

No = the threshold was not pre-specified

Unclear = information cannot be obtained from the paper

2.3 Risk of Bias: Could the conduction or interpretation of the index test have introduced bias?

high/ low/ unclear

High = at least one question is “no”, indicating that there is concern

Low = all questions are answered “yes”

Unclear = answer to at least one question is “unclear” and none are answered “yes”

(relating to questions 2.1. and 2.2.)

2.4 Concerns regarding applicability:

Are there concerns that the index test, its conduct, or interpretation differ from the review question?

high/ low/ unclear

High = minimally or non-invasive glucose monitoring was not performed adequately or correctly

Low =no such concerns

Unclear = information cannot be obtained from the paper

Additional relevant information:

**Domain 3: Reference Standard**

3.1 Is the reference standard likely to correctly classify the target condition?

yes/ no/ unclear

Yes = venous or arterial blood glucose (± capillary blood glucose) was used as reference standard for detection of hypoglycaemia

No = only capillary blood glucose was used as reference standard for detection of hypoglycaemia

Unclear = information cannot be obtained from the paper

3.2 Were the reference standard results interpreted without knowledge of the results of the index test?

yes/ no/ unclear

Yes = it is stated clearly that the individuals interpreting the reference test did not know the results of the index test, e.g. could not hear an alarm indicating hypoglycaemia

No = results of the index test were known by the individuals performing the reference test

Unclear = information cannot be obtained from the paper

3.3 Risk of Bias: Could the reference standard, its conduct, or its interpretation have introduced bias?

high/ low/ unclear

High = at least one question is “no”, indicating that there is concern

Low = all questions are answered “yes”

Unclear = answer to at least one question is “unclear” and none are answered “yes”

(relating to questions 3.1. and 3.2.)

3.4 Concerns regarding applicability:

Are there concerns that the target condition as defined by the reference standard does not match the review question?

high/ low/ unclear

High = the target condition was not hypoglycaemia, diagnosed via capillary/venous/arterial blood

Low = no such concerns

Unclear = information cannot be obtained from the paper

Additional relevant information:

**Domain 4: Flow and Timing**

4.1 Was there an appropriate interval between index test(s) and reference standard?

yes/ no/ unclear

Yes = reference test was performed in regular intervals (≤1h)

No = reference test were performed completely irregular/ in intervals (>1h)/ only if hypoglycaemia was suspected

Unclear = information cannot be obtained from the paper

4.2 Did all patients receive a reference standard?

yes/ no/ unclear

Yes = all patients who received the index test also received a reference standard in regular intervals

No = not all the patients who received the index test also received the reference standard

Unclear = information cannot be obtained from the paper

4.3 Did all patients receive the same reference standard?

yes/ no/ unclear

Yes = same reference standard was used for all patients and for all glucose values

No = different reference standards were used, e.g. different reference standard when hypoglycaemia was expected

Unclear = information cannot be obtained from the paper

4.4 Were all patients included in the analysis?

yes/ no/ unclear

Yes = no withdrawals or exclusions, or if those reasons are adequately explained

No = withdrawals or exclusions are not explained or accounted for

Unclear = information cannot be obtained from the paper

4.5 Risk of Bias: Could the patient flow have introduced bias?

high/ low/ unclear

High = at least one question is “no”, indicating that there is concern

Low = all questions are answered “yes”

Unclear = answer to at least one question is “unclear” and none are answered “yes”

(relating to questions 4.1., 4.2., 4.3 and 4.4)

Additional relevant information:

**Supplement 4**


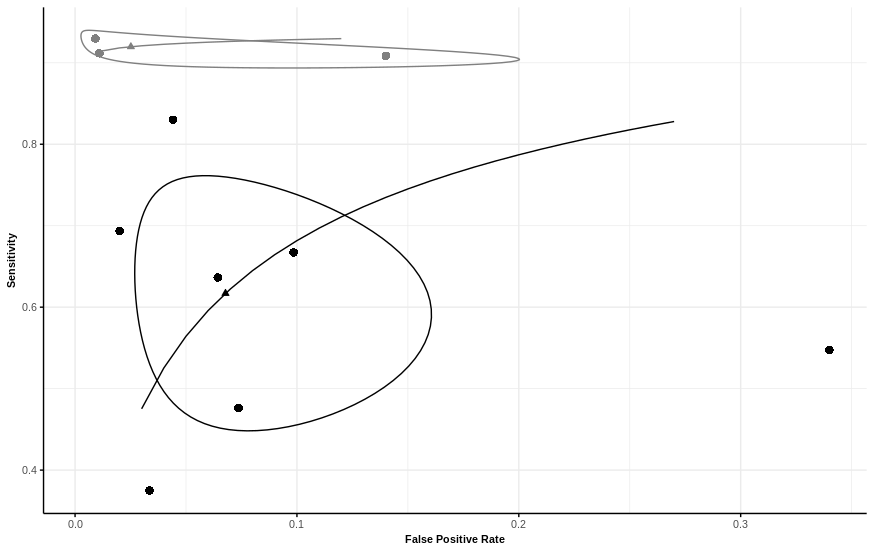


Summary receiver operating characteristic (SROC) curve for overall diagnostic accuracy to detect hypoglycaemia of MID and NID. SROC= solid curve, study data = circles, triangle = summary estimate, 95% confidence region = contour ellipsoid, black = low MARD, grey = high MARD). The pooled sensitivity was significantly higher in trials indicating a lower MARD (corresponding to a better overall performance).

**Supplement 5**


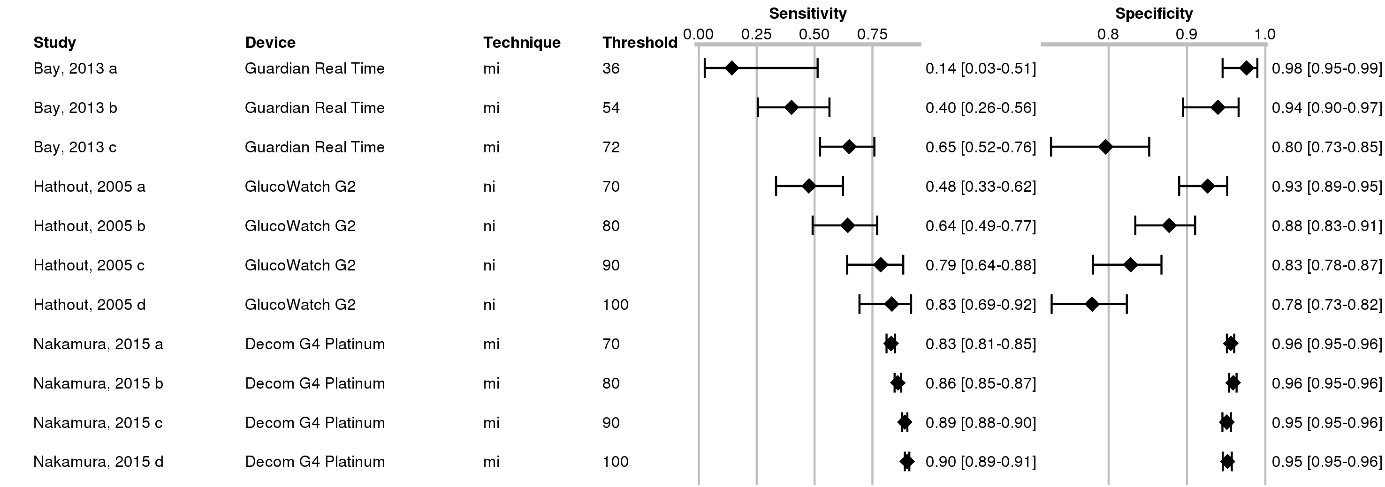


Forest plot of sensitivity and specificity with 95% confidence interval of MID and NID for detection of hypoglycaemia in studies applying different thresholds simultaneously. Threshold in mg/dl.
